# A Self-Cascade Penetrating Brain Tumor Immunotherapy Mediated by Near-Infrared II Cell Membrane-Disrupting Nanoflakes via Detained Dendritic Cells

**DOI:** 10.1021/acsnano.4c06183

**Published:** 2024-07-02

**Authors:** Bhanu Nirosha Yalamandala, Yu-Jen Chen, Ya-Hui Lin, Thi My Hue Huynh, Wen-Hsuan Chiang, Tsu-Chin Chou, Heng-Wei Liu, Chieh-Cheng Huang, Yu-Jen Lu, Chi-Shiun Chiang, Li-An Chu, Shang-Hsiu Hu

**Affiliations:** † Department of Biomedical Engineering and Environmental Sciences, National Tsing Hua University, Hsinchu 300044, Taiwan; ‡ Brain Research Center, National Tsing Hua University, Hsinchu 300044, Taiwan; § Department of Chemical Engineering, National Chung Hsing University, Taichung 402, Taiwan; ∥ Institute of Analytical and Environmental Sciences, National Tsing Hua University, Hsinchu 300044, Taiwan; ⊥ Department of Neurosurgery, Shuang Ho Hospital, Taipei Medical University, New Taipei City 23561, Taiwan; # Taipei Neuroscience Institute, Taipei Medical University, Taipei 11031, Taiwan; ∇ Department of Surgery, School of Medicine, College of Medicine, Taipei Medical University, Taipei 11031, Taiwan; ○ Institute of Biomedical Engineering, National Tsing Hua University, Hsinchu 300044, Taiwan; ◆ Department of Neurosurgery, Chang Gung Memorial Hospital, College of Medicine, Chang Gung University, Taoyuan 33305, Taiwan; ¶ College of Medicine, Chang Gung University, Kwei-San, Taoyuan 33302, Taiwan

**Keywords:** CuS nanoflakes, polymeric micelle, immunotherapy, immunoadjuvant, NIR II, brain tumor

## Abstract

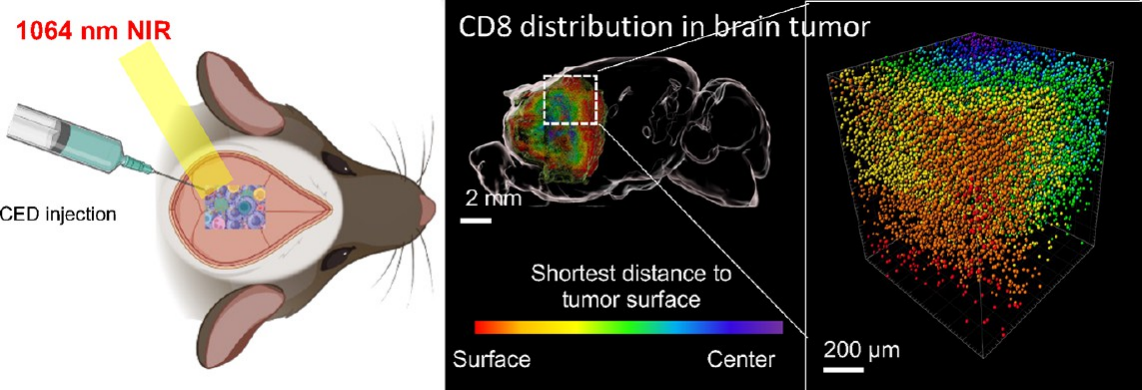

Immunotherapy can potentially suppress the highly aggressive glioblastoma (GBM) by promoting T lymphocyte infiltration. Nevertheless, the immune privilege phenomenon, coupled with the generally low immunogenicity of vaccines, frequently hampers the presence of lymphocytes within brain tumors, particularly in brain tumors. In this study, the membrane-disrupted polymer-wrapped CuS nanoflakes that can penetrate delivery to deep brain tumors via releasing the cell–cell interactions, facilitating the near-infrared II (NIR II) photothermal therapy, and detaining dendritic cells for a self-cascading immunotherapy are developed. By convection-enhanced delivery, membrane-disrupted amphiphilic polymer micelles (poly(methoxypoly(ethylene glycol)-benzoic imine-octadecane, mPEG-*b*-C18) with CuS nanoflakes enhances tumor permeability and resides in deep brain tumors. Under low-power NIR II irradiation (0.8 W/cm^2^), the intense heat generated by well-distributed CuS nanoflakes actuates the thermolytic efficacy, facilitating cell apoptosis and the subsequent antigen release. Then, the positively charged polymer after hydrolysis of the benzoic-imine bond serves as an antigen depot, detaining autologous tumor-associated antigens and presenting them to dendritic cells, ensuring sustained immune stimulation. This self-cascading penetrative immunotherapy amplifies the immune response to postoperative brain tumors but also enhances survival outcomes through effective brain immunotherapy.

## 1 Introduction

Glioblastoma (GBM), an exceptionally aggressive cancer, originates from glial cells and predominantly impacts the central nervous system (CNS).^1−6^ Standard treatment involves maximal safe resection followed by radiation/chemotherapy to improve survival. However, maximizing elimination of infiltrating malignant cells in GBM from a healthy brain tissue is unattainable, often resulting in frequent relapses.^7,8^ Additional chemotherapy is often hindered by brain endothelial cells, known as the tumor-associated blood–brain barrier (BBB), which reduces efflux transporters. Therefore, the BBB blocks approximately 99% of molecules with a molecular weight less than 500 Da.^9−11^ The BBB as a host organ experiences significant endogenous immunosuppression also posing challenges to immunotherapeutic strategies.^12−18^ Despite recent advances in immunotherapy, the impact of T cell infiltration into the brain during brain tumor treatment remains suboptimal. This is attributed to the immune privilege of the brain and the microenvironment of malignant glioma.^19,20^ In this regard, penetrating delivery has the potential to induce potent antitumor immunity.^21−24^

The cytotoxic T cells are often hampered by tumor heterogeneity constituted of high interstitial fluid pressure (IFP) and the presence of cancer-associated fibroblasts. They limit transport and immune escape of therapeutic agents, especially in the brain.^25−28^ To address these issues, one potential strategy is to use specific particles to create gaps between cancer cells, called nanoparticle-induced endothelial leakage (NanoEL).^29−31^ It induces extravasation of cancer cells into the surrounding vascular areas. The NanoEL gaps also allowed for nanomedicine to pass through, which has been reported recently.^31−34^ It is intended to kill tumors, but it may also inadvertently cause leakage in tumor vasculature, thereby lowering the intravascular barrier for viable cancer cells to enter the circulation.^33,34^ Furthermore, utilizing NanoEL gold particles leads to increased leakage of larger sized nanoparticles and could bring about complete regression of primary tumors and attack against the secondary metastatic tumor when it is at the micrometastasis stage.^29^ Another recently reported study on amyloid-induced endothelial leakage (APEL) in human microvascular endothelial cells exposed to Aβ42 oligomers, fibrils, and amyloid-seeded nanoparticles is reminiscent of NanoEL.^35^

An alternative strategy is to employ membrane-disrupting mechanisms involving nanoparticles that are quiescent at neutral pH and selectively disrupt cancer cell membranes upon exposure to an acidic tumor microenvironment (pH 6.4–6.8).^36^ In this regard, pH-sensitive nanoparticles enhance tumor permeability, prolong circulation in the blood of polyzwitterionic drug conjugates with cell membrane affinity, and disrupt cell junctions. In glioma immunotherapy, a hybrid “cluster bomb” nanovaccine with a high antigen loading capacity on a zinc oxide (ZnO) surface acted as a stimulator of dendritic cells (DCs) and activated T cells.^37^

The second near-infrared biological window (NIR II, 950–1350 nm) is characterized by reduced photon scattering and enhanced penetration depth, proving the wide attraction in biomedical applications.^38,39^ Various NIR II photosensitizers have been used, including ICG complexes and Se-doped nanoparticles (NPs), but challenges remain in achieving high singlet oxygen quantum yields and optimal therapeutic efficacy.^40−42^ Due to the low toxicity and significant photothermal conversion efficiency, copper sulfide (CuS) nanomaterials exhibiting strong absorbance in the NIR II window have been studied for photothermal therapy (PTT).^43^ Recent observations exhibit the excellent efficacy of CuS nanoparticles for type I photodynamic therapy (PDT), demonstrating their ability to generate highly reactive oxygen species (ROS) to eliminate cancer cells upon an NIR II exposure.^44^ Therefore, there is an urgent need to design ultrasmall CuS nanoparticles capable of deeper tissue penetration to improve the effectiveness of PTT and PDT applications.

**Figure 1:**
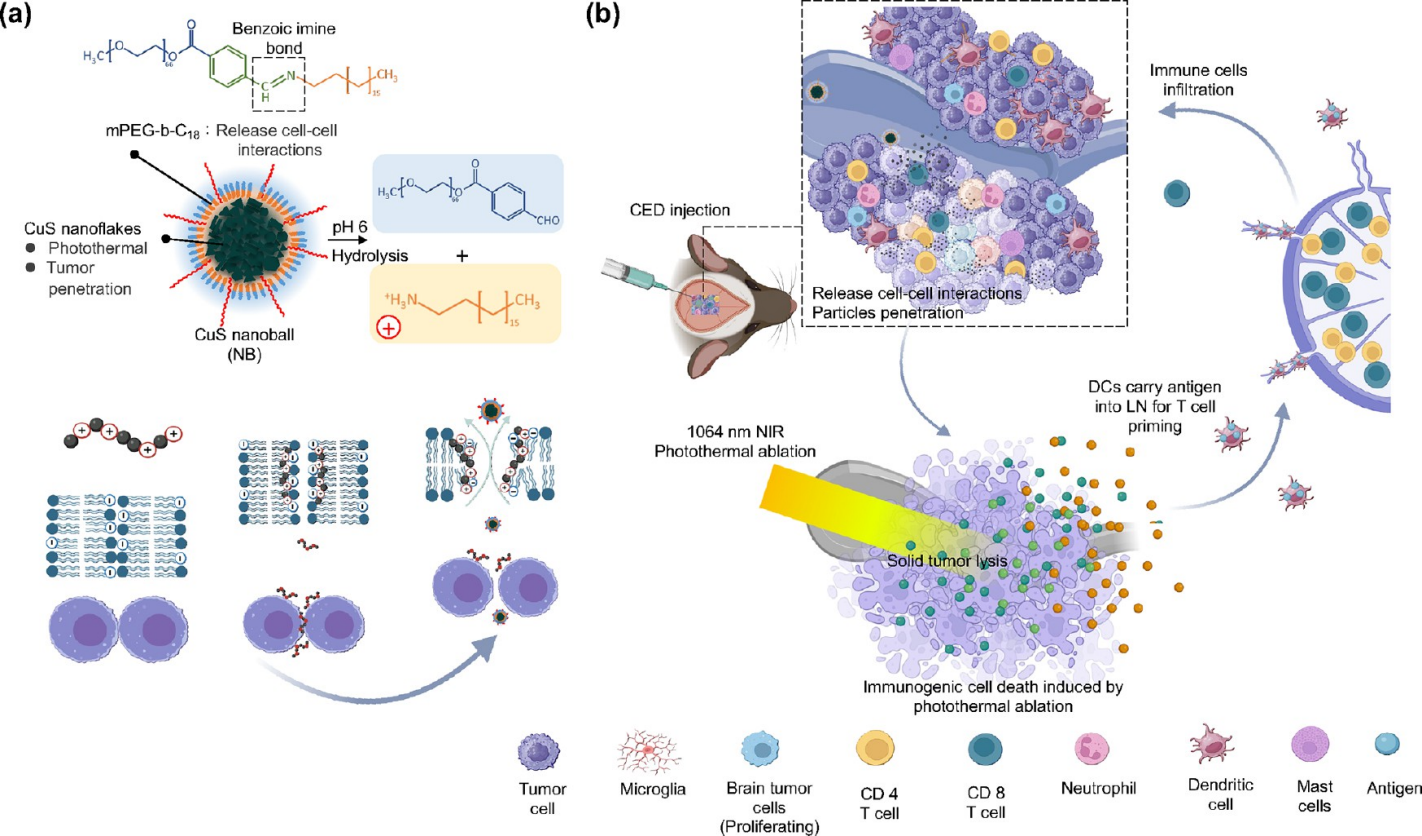
Schematic illustration of preparation and features of the CuS nanoball (NB) composed of membrane-disrupted polymer-wrapped CuS nanoflakes for brain immunotherapy. (a) The disruption of the benzoic-imine bond in mPEG-*b*-C18 on CuS NB enhances tumor permeability, reaching deep brain tumors by releasing cell–cell interactions under weak acidic conditions. (b) CuS NB is efficiently accumulated in brain tumors through continuous positive pressure infusion of CED. Membrane disruption-mediated tumor penetration and low-power NIR II irradiation (0.8 W/cm^2^) resulted in CuS nanoflakes generating intense NIR II-generated heat deep within the tumor, promoting antigen release. This process preserves autologous tumor-associated antigens and presents them to dendritic cells, amplifying CD4^+^ and CD8^+^ T cell-mediated immune responses.

Here, a self-cascade penetrating brain tumor immunotherapy mediated by near-infrared II cell membrane-disrupting nanoflakes was developed. This system consists of a membrane-disrupting polymer (poly(ethylene glycol)-benzoic imine-octadecane, mPEG-*b*-C18) loaded with CuS nanoflakes, termed CuS nanospheres (CuS NBs). *Via* a convection-enhanced delivery system (CED) at the brain tumor, the charge conversion of CuS NB enhances tumor permeability to reach deep brain tumors by releasing cell–cell interactions under weak acidic conditions (Figure 1a). Furthermore, CuS also induced NanoEL at the tumor site. Then, CuS NB accumulates in the brain tumor and distributes in a deep tumor area upon low-power NIR II irradiation (0.8 W/cm^2^). At the tumor site, NIR II hyperthermia through CuS nanosheets promotes cancer cell apoptosis and promotes the release of tumor-associated antigens (TAA). These TAAs are then captured by primary amine groups on NB, which also serve as a transporter of antigens for immunogenic cell death. The *in situ* capture system further promotes the retention of antigen release to achieve sustained immune stimulation and brain tumor suppression. The captured antigen further recruits more dendritic cells to enhance the immune response of CD4^+^ and CD8^+^ T cells. Therefore, the proposed antigen capture mechanism shows great potential for application in enhancing cancer immunotherapy.

## 2 Results and Discussion

### 2.1 Synthesis of CuS NBs

**Figure 2:**
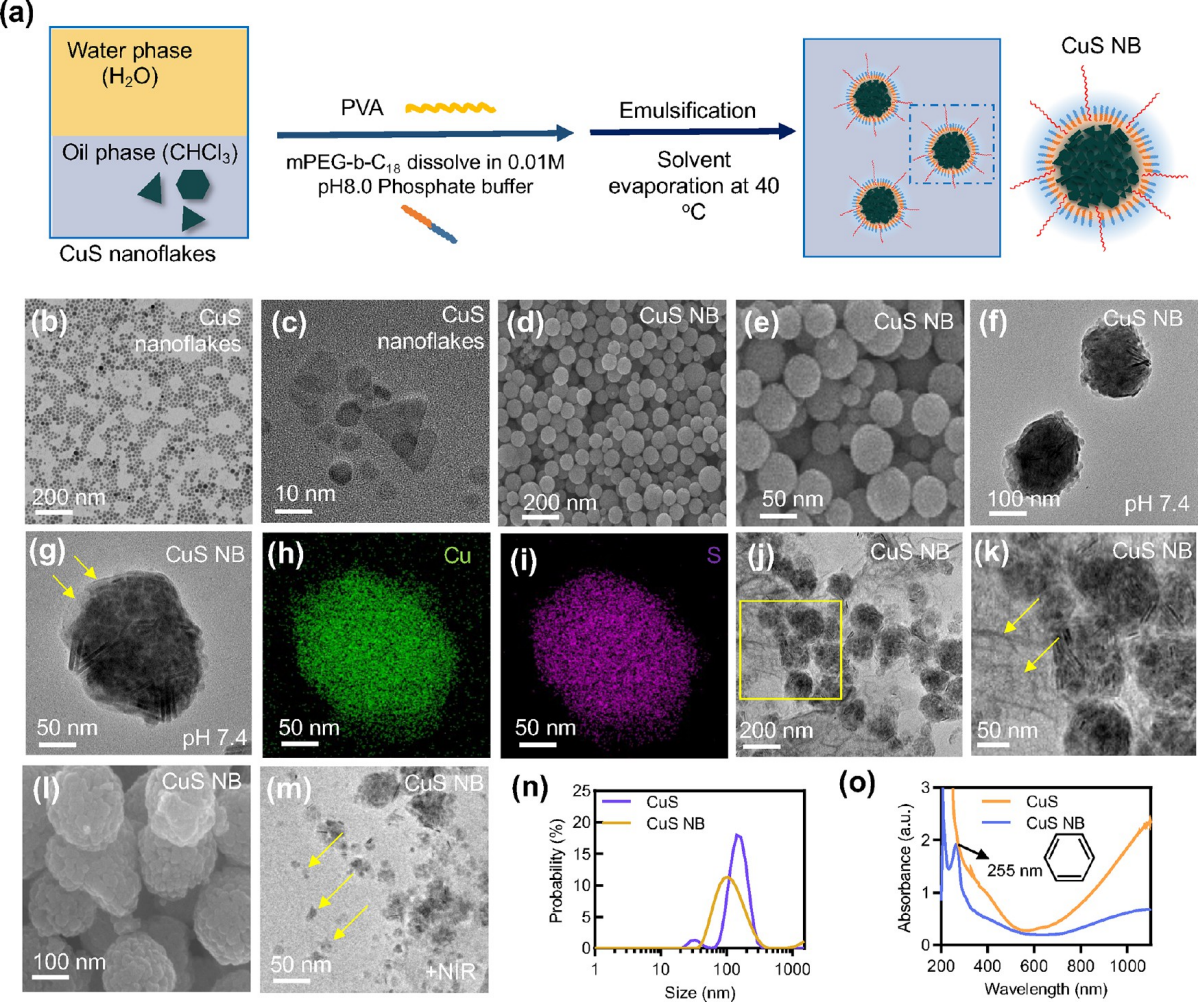
Synthesis and characterizations of CuS NB (mPEG-*b*-C_18_@CuS). (a) Schematic representation showing the synthesis of CuS nanoballs composed of a hydrophilic methoxy poly(ethylene glycol) (mPEG) moiety and a hydrophobic octadecane chain (C_18_) with a pH-sensitive benzoic-imine linker. (b,c) TEM images of CuS nanoflakes. (d,e) SEM and (f,g) TEM images of CuS NBs. (h,i) Elemental mapping analysis of CuS NB. (j–l) SEM and TEM images of CuS NB at pH 6. (m) TEM images of CuS NB after NIR irradiation. (n) UV–visible spectrum of CuS nanoflakes and CuS NB. (o) Size distribution of CuS nanoflakes and CuS NB.

The synthesis process of CuS NB composed of CuS nanoflakes and mPEG-*b*-C18 is depicted in Figure 2a. Initially, CuS nanoflakes were synthesized using the hot-injection method, wherein a room-temperature *S*-oleylamine solution was rapidly injected into a preheated CuCl_2_ mixture solution. During the preheated stage, CuCl_2_ coordinated with oleylamine and oleic acid through their amino group and the C=C double bond, respectively. Subsequently, the hot injection into the Cu precursor facilitated the generation of various active S substances that contributed to the growth of CuS. The temperature drops in the mixture during the injection stage helped limit the overlap of nucleation and growth times, resulting in a uniform size distribution of the CuS nanoflakes.^45^

To form the CuS NB, the emulsion process was applied by using both poly(vinyl alcohol) (PVA) and mPEG-*b*-C18 to stabilize the CuS nanoflakes. In brief, the oil-soluble CuS nanoflakes were dissolved in chloroform, and a mixture containing 0.5 wt % PVA and mPEG-*b*-C18 was introduced into deionized water. Subsequently, the organic solvent was removed by applying sonication and solvent evaporation at 40 °C. During this process, the emulsion method, utilizing PVA/mPEG-*b*-C18 as a binder, was employed, leading to the uniform assembly and dispersion of CuS nanoflakes throughout the PVA/mPEG-*b*-C18 nanomatrix. This observed uniformity is attributed to potential interactions, such as hydrogen bonding or dipole–dipole interactions, between the functional groups on the surface of CuS nanoflakes and the hydroxyl groups of the polymers.

By systematically examining various PVA/mPEG-*b*-C18 ratios, Table S1 illustrates that alternative preparation ratios exhibit excellent stability in appearance and display monodispersity according to dynamic light scattering (DLS) analysis. TEM observations reveal a spherical morphology for all three samples. Increasing amounts of mPEG-*b*-C18 correspond to larger CuS nanoballs in transmission electron microscopy (TEM) images, aligning with DLS findings. However, in the group with a reduced PVA concentration (0.1 wt %), noticeable agglomeration and precipitation occurred postsynthesis. In contrast, the PVA-only group displayed a satisfactory size distribution in DLS measurements, albeit with a less uniform morphology than other groups. The choice of 0.5% PVA for the CuS nanoballs was validated after these tests. We further hypothesized that the mechanism of formation relies on the hydrophobic interaction between CuS nanoflakes and the hydrophobic carbon chain moiety of mPEG-*b*-C18, forming a hydrophobic core with a hydrophilic outer layer stabilized by PVA molecules. Therefore, as the quantity of mPEG-*b*-C18 increases, larger CuS nanoballs are observed.

### 2.2 Morphology and Size Distribution of CuS NBs

To investigate the morphologies of the resulting nanoparticles, transmission electron microscopy (TEM) and scanning electron microscopy (SEM) were employed. TEM images in Figure 2b,c revealed that CuS nanoflakes exhibit platelet-like morphologies with diverse shapes and sizes, approximately 20 nm in dimension. The growth pattern of these CuS nanoflakes may be attributed to the intrinsic hexagonal crystallographic structure of covellite, wherein nanoparticles tend to grow slowly along the (001) directions and rapidly in other directions.^46,47^ SEM images illustrate the morphologies of the resulting CuS NBs in Figure 2d,e. Following the formation of CuS NBs, as depicted in Figure 2f,g, the CuS nanoflakes assembled to display a spherical conformation with an approximate diameter of 180 nm, as observed through TEM. Moreover, the polymer outer layer of CuS NBs and the core of CuS nanoflakes could be discerned in the TEM images. The white arrow indicates the outermost polymer layer. At the same time, CuS nanoflakes are marked by a yellow arrow (Figure 2f). Elemental mapping further confirmed the successful encapsulation of CuS nanoflakes (Figure 2h,i).

The pH-responsive polymer, mPEG-*b*-C18, consists of a hydrophilic methoxy poly(ethylene glycol) (mPEG) moiety and a hydrophobic octadecane chain (C18) with a pH-sensitive benzoic-imine linker that undergoes partial hydrolysis in the extracellular pH environment of solid tumors. To preliminarily assess the morphological effects of the pH, TEM images of CuS NBs were obtained after treatment with a pH 6 buffer solution. As illustrated in Figure 2j,k, some polymers on the NBs were dissolved, forming a surrounding layer and free polymer chains (as indicated by yellow arrows). In Figure 2l, SEM observation revealed a roughened surface of the NBs, indicative of the loosening of the polymer chains. Upon NIR irradiation, CuS nanoflakes demonstrate pronounced absorption, triggering a series of energy conversion events that culminate in localized heating within the core (Figure 2m). This rise in temperature induces a transition in the coating polymer and kinetic energy of CuS nanoflakes, facilitating the release of the encapsulated nanoflakes. Freed from their confines, the nanoflakes, driven by thermal expansion and ensuing pressure accumulation, exhibit heightened photothermal ablation capabilities.

Dynamic light scattering (DLS) was employed to assess the size distribution analysis. As illustrated in Figure 2n, unmodified CuS exhibited a size of approximately 450 nm, indicative of aggregation and poor dispersity in water. In contrast, CuS nanoballs displayed a diameter of around 250 nm, slightly larger than observed in the TEM image, owing to the presence of the mPEG-*b*-C18 outer layer and the hydrodynamic diameter. Furthermore, a UV–vis spectrometer was utilized to examine the adsorption spectrum of the two nanoparticles. As depicted in Figure 2o, CuS nanoflakes exhibited a broad peak spanning 800 to 1100 nm, demonstrating strong absorption in the near-infrared (NIR) window. The absorption profile of CuS nanoballs around the NIR window remained essentially unchanged after the encapsulation of CuS nanoflakes. However, a distinct peak at 255 nm emerged, likely associated with the benzene structure of mPEG-*b*-C18, providing further confirmation of the successful production of CuS nanoballs.

### 2.3 Physicochemical Characterization of CuS NBs

**Figure 3:**
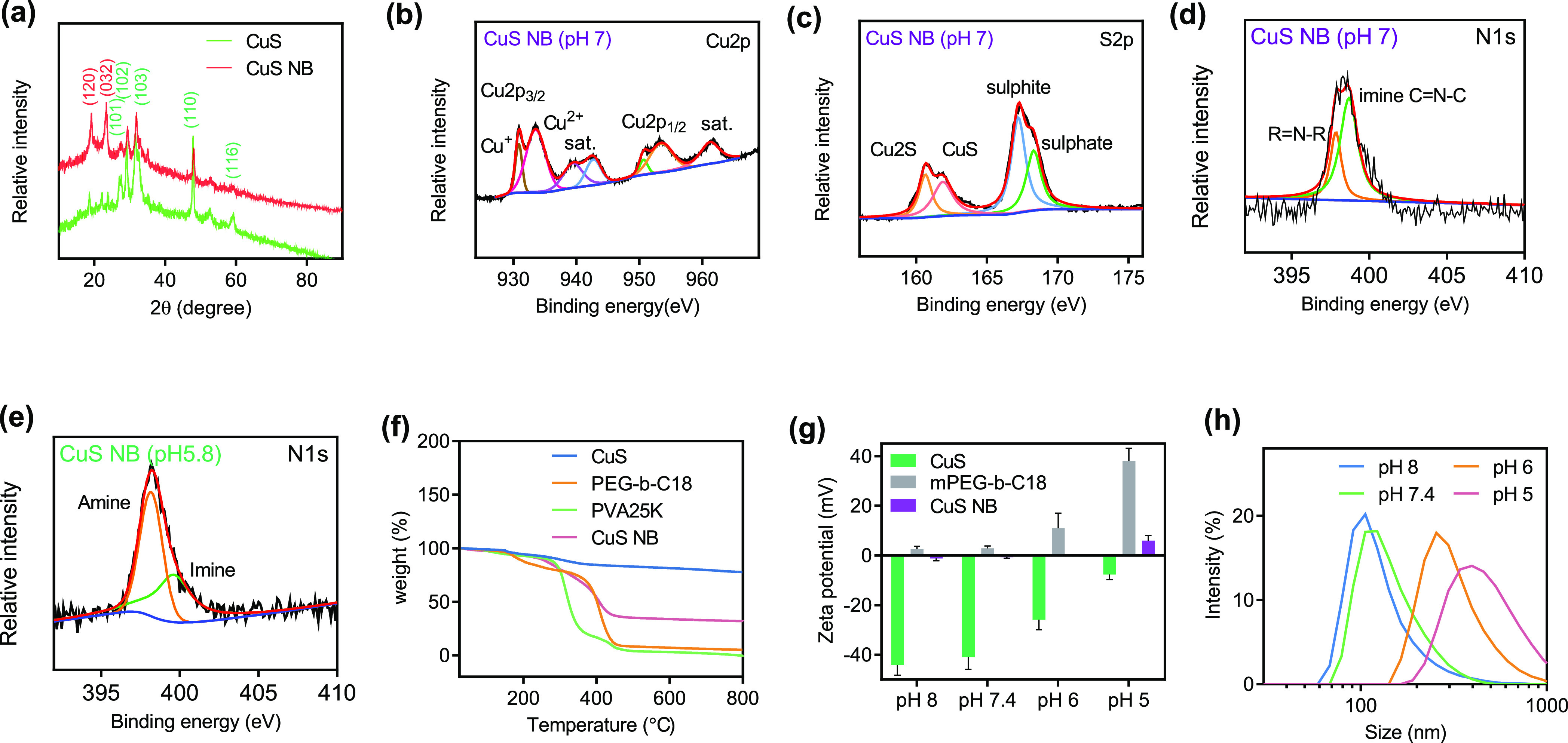
Physicochemical characterization of CuS NB. (a) XRD spectra of CuS nanoflakes and CuS NB. (b–d) XPS spectra of all the elements, Cu 2p, S 2p, and N 1s spectra of CuS nanoballs. (e) XPS N 1s spectra of CuS nanoballs at pH 5.8. (f) TGA analysis of CuS nanoflakes, CuS nanoballs, PVA25K, and mPEG-*b*-C_18_. (g) Zeta potential and (h) size distribution of CuS nanoballs under various conditions.

X-ray diffraction (XRD) analysis was performed to discern the crystalline features (Figure 3a). The diffraction peaks observed at 27, 29, 32, and 48° corresponded to the crystal planes of (101), (102), (103), and (110), respectively.^45^ Upon assembly into CuS NBs, the intensity of the diffraction signals from CuS nanoflakes diminished due to the presence of the amorphous polymer coating. To further explore the cleavage of the benzoic-imine bond, X-ray photoelectron spectroscopy (XPS) was employed for analysis, and the results are presented in Figure 3b–e. Seven distinct peaks were observed in the Cu spectrum, corresponding to Cu 2p_3/2_, Cu 2p_1/2_, and satellite peaks. The S spectrum exhibited two major peaks: the first, around 162 eV, denoted the bonding of CuS and Cu2S, while the second, around 167 eV, indicated the presence of sulfite and sulfate. The analysis revealed that the synthesized CuS nanoflakes contained CuS and Cu2S, along with sulfur oxides (sulfites and sulfates) likely formed during the synthesis.

Upon comparing the N 1s spectrum of CuS nanoballs at pH 7 and pH 5.8, distinct peaks corresponding to R=N–R and C=N–C groups were observed at binding energies of 398.0 and 398.9 eV, respectively. Notably, the imine group peak weakened in acidic conditions, while the amine peak strengthened (Figure 3d,e). The XPS spectra of Cu 2p and S 2p of CuS nanoballs at pH 5.8 exhibited patterns similar to those at pH 7 (Figure S1a,b). The C 1s XPS spectra indicated the formation of C–NH functional groups, suggesting the breakage of imine bonds (Figure S1c,d). In the pH 5.8 environment, the presence of C–NH and C=O bonds further confirmed the cleavage of the benzoic-imine bond and the emergence of amino groups (Figure 3e).

To analyze the composition of the nanoparticles and assess their thermal stability, thermogravimetric analysis (TGA) was employed. In Figure 3f, all four materials exhibited only a 1–2% weight loss in the temperature range of 30 to 100 °C, attributable to the evaporation of water or solvent molecules. This suggests that all the materials maintained good thermal stability within the typical hyperthermia temperature range of 40 to 45 °C. Notably, CuS nanoballs displayed a significant weight loss between 100 and 400 °C, indicating the removal of polymer and impurities associated with CuS. The detailed weight loss profiles are presented in Table S2. The organic-to-inorganic ratios were estimated using TGA results, revealing that approximately ∼24% of CuS nanoflakes were encapsulated.

The pH-responsive polymer, mPEG-*b*-C18, features a pH-sensitive benzoic-imine linker that undergoes partial hydrolysis in the extracellular pH environment of solid tumors (Figure S2). Following hydrolysis, the surface charge transitions from neutral to positive due to the emergence of amino groups after linker cleavage. The surface zeta potential was measured to monitor the hydrolysis of the benzoic-imine linker. In Figure 3g, mPEG-*b*-C18 exhibited a neutral charge in physiological and weak basic environments, with the zeta potential increasing at pH 6 and 5, representative of the tumor and endosome environments. The elevation of the zeta potential from 2 to 11 and 40 mV confirmed the hydrolysis of the benzoic-imine linker and the presence of amino groups. However, the change in the zeta potential was not observed in CuS nanoballs under acidic conditions. This phenomenon may be attributed to the electrostatic interaction between the negative CuS nanoplates, neutralizing the surface potential. Furthermore, dynamic light scattering (DLS) measurements were also conducted to examine the size distribution changes of CuS nanoballs in different pH environments. At weak acidic conditions, the size slightly increased, as depicted in Figure 3h.

### 2.4 Photothermal Efficacy and *In Vitro* Study

**Figure 4:**
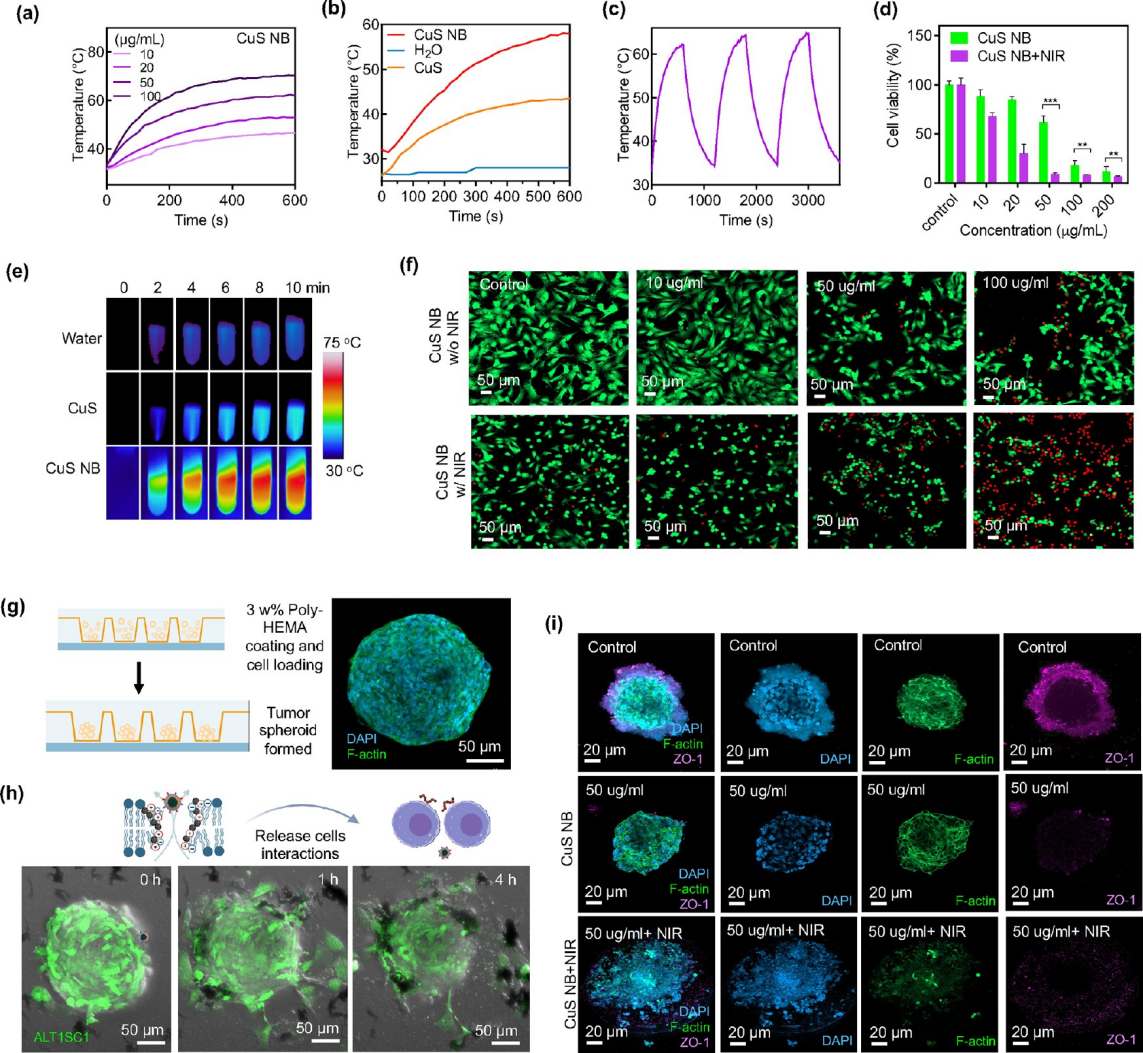
Photothermal conversion and *in vitro* study. (a) Thermal heating profiles of CuS NB at various concentrations. (b) Thermal heating profiles of CuS nanoflakes and CuS NB. (c) Photothermal conversion test. (d) Cell viability of ALTS1C1 treated with CuS NB and CuS NB+NIR. (e) Infrared thermal imaging of water, CuS, and CuS nanoballs (*n* = 4; mean ± s.d.; **p < 0.01; ***p < 0.05; one-way ANOVA with Tukey’s multiple-comparison tests). (f) CLSM images of live/dead staining of ALTS1C1 cells with or without NIR irradiation at different concentrations of CuS NB. (g) Schematic representation of 3D tumor spheroid formation. (h) CLSM images of the ALTS1C1-GFP tumor spheroid cocultured with 100 μg mL^–1^ CuS nanoballs over time. (i) CLSM images depicting ALTS1C1 tumor spheroids subjected to various treatments. Cell nuclei, cytoskeleton, and ZO-1 were visualized through staining with DAPI (blue), F-actin (green), and a primary antibody (violet).

The photoconversion experiments were conducted to investigate the photothermal effect of the nanoparticles. The temperature increment of the CuS nanoballs exhibited a concentration-dependent temperature pattern (Figure 4a). The temperature of CuS nanoballs could rise to 70 °C after 10 min of 1064 nm NIR irradiation (0.8 W/cm^2^) at the concentration of 100 μg/mL. However, the temperature of water only had a 2 °C increment after the same treatment. In addition, CuS NB exhibited a higher temperature rise compared with bare CuS nanoflakes, which may be related to the different dispersibility of the two nanoparticles in water (Figure 4b). After three cycles of 1064 nm NIR irradiation and cooling, the photothermal effect of CuS nanoballs remained good, indicating the prospective of CuS nanoballs in photothermal therapy (Figure 4c).

The *in vitro* antitumor activity was assessed using a LIVE/DEAD viability/cytotoxicity kit and a cell viability assay. ALTS1C1 cells were subjected to varying concentrations of CuS NB and incubated for 2 h followed by irradiation with a 1064 nm NIR laser for 5 min. Postirradiation, a significant decrease in cell viability was observed compared to the group without NIR irradiation (Figure 4d). Infrared thermal imaging of water, CuS, and CuS NB at 100 μg/mL further highlighted the photothermal effects (Figure 4e). Additionally, we analyzed the particle size distribution after 1 min of irradiation with a 1064 nm NIR laser at different pH values. The results, depicted in Figure S3, demonstrated a leftward shift in the distribution of CuS nanoballs after irradiation at pH 8, 7.4, and 6. This shift suggests a minor alteration in the structure of the CuS NB following NIR irradiation.

### 2.5 3D Tumor Spheroid Model

While two-dimensional (2D) culture remains the conventional method for *in vitro* experiments, it possesses inherent limitations, notably its inability to accurately replicate the intricate interactions between cells and the extracellular matrix. Such drawbacks hinder its capacity to represent the complexities of *in vivo* situations. Our study employed a three-dimensional (3D) tumor spheroid culture to better emulate the tumor environment. ALTS1C1 cells were cultured in a dish equipped with microchips within the well (Figure 4g). These microchips featured micrometer-sized holes into which cells fell under gravity when introduced as a cell suspension. A poly(2-hydroxyethyl methacrylate) (poly-HEMA) coating on the microchips prevented cell adhesion to the bottom, promoting the aggregation of cells within the holes to form spheroids. After 2–3 days of cultivation, the ALTS1C1 spheroids were utilized for subsequent experiments.

To elucidate the impact of CuS NBs in a 3D tumor environment, we initially cocultured ALTS1C1-GFP tumor spheroids with 50 μg/mL CuS NB, conducting prolonged observations. In Figure 4h, at 0 h, the tumor spheroid exhibited robust green fluorescence, with some surrounding debris, possibly indicative of dead cells. After adding particles for 1 h, the fluorescent signal gradually faded, and some tumor spheroids underwent deformation and cell release, suggesting a reduction in cell interaction. By the fourth hour, the GFP signal from the outer layer of the spheroid vanished, and particle accumulation on the spheroids was evident. Furthermore, the structural integrity became compromised, indicating the destructive and cytotoxic effects of CuS nanoballs on tumor spheroids.

A similar experiment was conducted with a lower concentration of CuS nanoballs. For tracking purposes, the nanoparticles were labeled with Cy5.5 (Figure S4). After the addition of 20 μg/mL CuS NB, nanoparticles with violet fluorescence adhered to the outer surface of ALTS1C1 tumor spheroids by 1 h. As time progressed, the GFP signal entirely disappeared by the fifth hour, indicating cell death slightly earlier than that of the high-concentration group, possibly due to the smaller size (∼100 μm) of the spheroids in this group. These results suggest that CuS nanoballs demonstrate potent antitumor efficacy in 2D *in vitro* experiments and 3D tumor spheroids.

In addition to tumor penetration, we examined cell–cell interactions through zonula occludens-1 (ZO-1) staining, also known as tight junction protein-1. The untreated spheroids exhibited a solid and dense morphology, with apparent ZO-1 staining on the surface. Under a higher resolution, a slightly foggy outer layer was observed (Figure 4i). This could be attributed to poor staining of DAPI or the aggregation of other small cell clusters. Additionally, no ZO-1 signal was observed in the inner region of the spheroid, possibly due to the relatively short culture time, where tight junctions between cells were not fully developed.

Compared to the control group, spheroids treated with 50 μg/mL CuS nanoballs showed a less dense structure, with a looser morphology and dead cells nearby. The ZO-1 signal was weaker, particularly at a higher resolution, indicating the disruptive effect of the CuS nanoballs on the spheroid. Notably, the outer layer of dead cells was not visible at a higher resolution, possibly due to the spheroid transfer from microchips to a confocal dish through suction and pipetting. To further investigate photothermal ablation in the 3D tumor spheroid model, we irradiated the spheroids with 1064 nm NIR after coculturing with 50 μg/mL CuS nanoballs for 2 h. Postirradiation, the spheroids lost their compact structure, and the presence of ZO-1 could not be observed compared to the control group.

The synthesized CuS nanoballs demonstrated inherent membrane-disrupting and extracellular matrix (ECM)-destructive abilities, attributed to the outer mPEG-*b*-C18 layer, as evidenced by both 2D and 3D *in vitro* examinations. The disruption of the lipid bilayer composition in cells can be attributed to the amphiphilic nature of mPEG-*b*-C18 and its capacity to interact with lipid molecules. The hydrophilic mPEG (polyethylene glycol) block tends to interact with water molecules, while the hydrophobic C18 (octadecyl) block prefers to interact with the hydrophobic tails of lipid molecules within the bilayer.^48,49^ This amphiphilic behavior allows mPEG-*b*-C18 to insert itself into the lipid bilayer, leading to disruption of the bilayer’s organization and stability. Consequently, this disruption compromises the integrity of the lipid bilayer, impacting essential cellular processes, including membrane permeability, signaling, and cell–cell interactions.

To discern whether the reduction in ZO-1 expression resulted from tumor cell death rather than direct evidence of membrane disruption, we employed live and dead cell staining. As demonstrated by CLSM images, most cells remained viable after treatment with mPEG -*b*-C18 or CuS NB (Figure S5). Upon NIR application to the CuNB-treated group, some cell death could be induced. Coupled with the image depicted in Figure 4i, it becomes evident that CuS NBs alone can diminish the expression of ZO-1. The CuS nanoflake core also exhibited a potent photothermal ablation ability upon irradiation with a 1064 nm NIR laser. These properties underscore the potential of CuS nanoballs in tumor therapy, where the polymer outer layer may facilitate nanoparticle penetration and disrupt solid tumors, followed by photothermal therapy to eliminate residual tumor cells.

To further assess the NanoEL of CuS NB, we employed transepithelial electrical resistance (TEER) and Transwell inserts to gauge cell leakage within cell culture models utilizing microvascular endothelial cells (bEnd.3 cells) and endothelial cells (human umbilical vein endothelial cells (HUVECs)). bEnd.3 and HUVECs cells were cultured on Transwell inserts for 48 h to foster the development of high-density cell sheets. Subsequently, 20 μg/mL CuS NBs were introduced into the system (Figure S6a). The decline in TEER values was observed at 6 and 24 h post-treatment with particles in a Transwell, indicative of diminished cell interactions (Figure S6a and S6b). CLSM images further revealed some leakage from the cell sheets following particle coincubation.

### 2.6 Cytotoxicity, Cellular Membrane Leakage, and Cell Uptake

**Figure 5:**
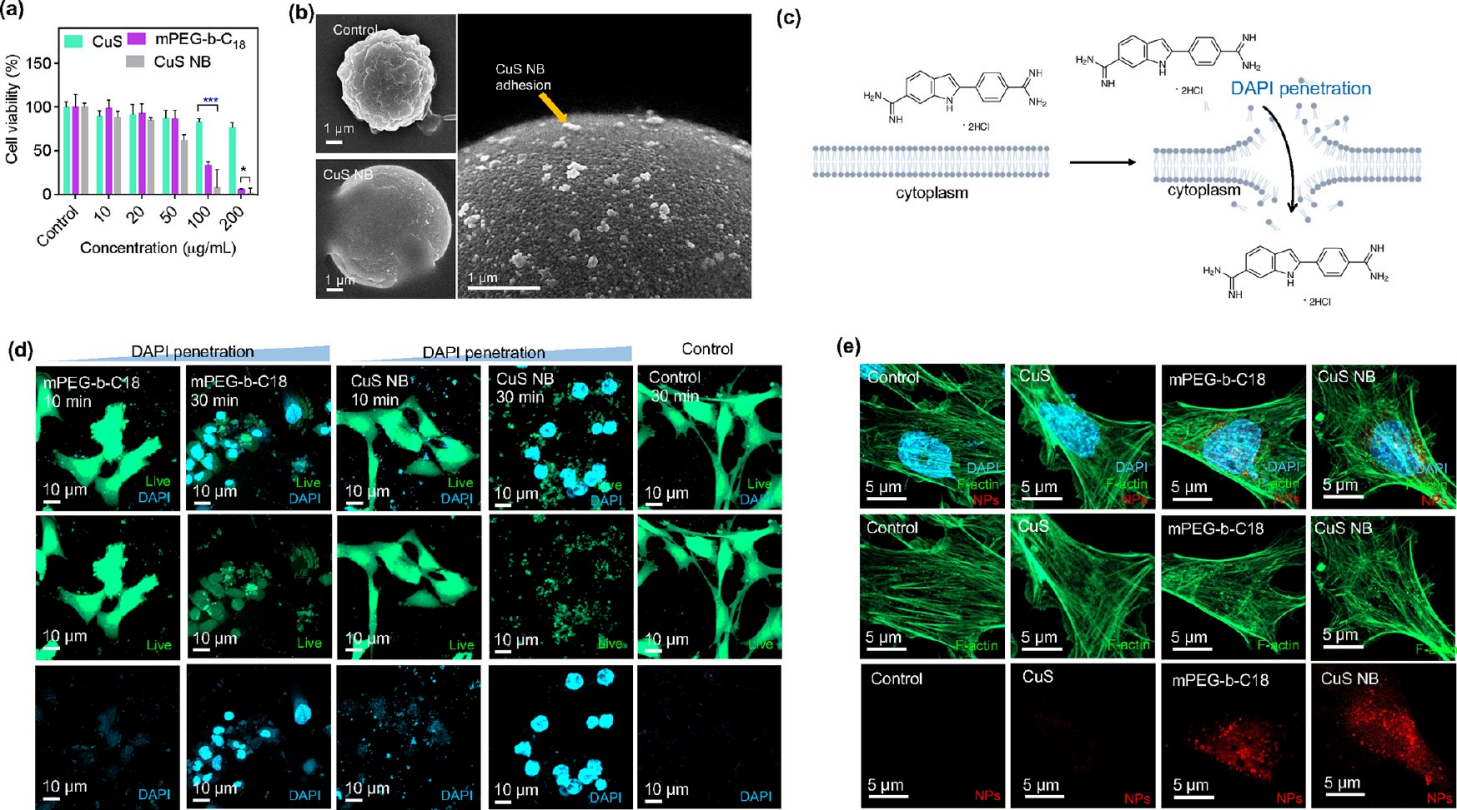
Cellular membrane leakage and cell uptake. (a) Cell viability of ALTS1C1 treated with CuS nanoflakes, mPEG-*b*-C_18_, and CuS nanoballs at various concentrations (*n* = 4; mean ± s. d.; *p < 0.05; ***p < 0.05; one-way ANOVA with Tukey’s multiple-comparison test). (b) SEM images of ALTS1C1 cells treated by CuS NB. (c) Graphical representation of cell membrane impermeability. (d) Examination of membrane disruption in CuS nanoballs at varying concentrations using DAPI staining visualization and a LIVE cytotoxicity kit for assessing membrane disruption indicators and cell morphology, respectively. (e) Concentration-dependent cellular uptake of CuS nanoflakes, mPEG-*b*-C18 micelles, and CuS NB. The cell nucleus, cytoskeleton, and CuS nanoballs were stained with DAPI (blue), F-actin (green), and cy5.5 (red), respectively.

The cytotoxicity of two nanoparticles was assessed using two cell lines, ALTS1C1 (murine astrocytoma cell line) and NIH3T3 (murine normal fibroblast cells), employing the PrestoBlue assay. Each cell line was exposed to varying concentrations of CuS nanoflakes, mPEG-*b*-C18, and CuS NB for 1 day. As depicted in Figure 5a, CuS nanoflakes alone demonstrated good biocompatibility, with cell survival rates reaching up to 76 and 79% in both ALTS1C1 and NIH3T3 cell lines, even at the highest concentrations. In contrast, the cell viability of the polymer mPEG-*b*-C18 drastically decreased, reaching 34 and 14% in ALTS1C1 and NIH3T3, respectively, when compared to CuS nanoflakes. Similarly, after the assembly of CuS NB, a significant reduction in the cell viability was observed for each cell line. This suggests that the substantial drop in cell viability may be attributed to the nonselective toxicity of mPEG-*b*-C18.

To assess the interactions between CuS NB and cells, SEM was employed to observe the cell morphology at various time points during treatment with 20 μg/mL CuS NB. The SEM images in Figure 5b reveal the attachment of NB to the cell membrane surface, indicating robust interactions with the cells. In accordance with the existing literature, Kuroda et al. presented an approach to comprehend particle-induced cellular membrane leakage, utilizing DAPI as a marker for membrane permeability.^50^ DAPI, a commonly used dye for staining fixed cell nuclei, binds strongly to the A-T-rich regions of nucleic acids and selectively stains cells with high permeability (Figure 5c). In this study, we utilized DAPI to detect changes in cell membrane permeability, which were observed through a confocal microscope. Figure 5d illustrates the detection of DAPI signals after a 30 min incubation for both mPEG-*b*-C18 and CuS NB groups, indicating molecular effects on cell membranes. The results indicated that both mPEG-*b*-C18 and CuS NB demonstrated efficacy in inducing cellular membrane leakage compared to the control group. This effect can be attributed to PEG’s hydrophilic nature and the carbon chain’s hydrophobic nature, which may disrupt the structure of the cell membrane composed of a lipid bilayer. This disruption leads to cell morphological fragmentation and subsequent cell death.

In an in-depth study of the impact of nanoparticles on cell phagocytosis, we conducted cellular uptake experiments. ALTS1C1 cells were subjected to varying concentrations of either CuS nanoflakes or CuS NB, stained with rhodamine B isothiocyanate (RITC) and Cy5.5, respectively, and then incubated for a day. Figure 5e illustrates the substantial accumulation of CuS NB compared to other groups, suggesting a potent cell uptake efficacy associated with cell leakage effects. For a more detailed examination, the group treated with CuS nanoflakes exhibited a disordered cytoskeleton. At 100 and 200 μg/mL, most cells displayed a fragmented morphology, indicative of cell death under microscopic observation (Figure S7). Furthermore, to track the temporal patterns of cellular uptake of CuS nanoballs, ALTS1C1 cells were treated with 100 μg/mL CuS nanoballs and incubated for 1, 2, 3, and 4 h. As depicted in Figure S8, cells initiated uptake of CuS nanoballs after 1 h, with uptake intensifying over prolonged incubation periods. However, alterations in the cell morphology were noticeable after 1 h of incubation and progressed to fragmentation after 3 h, mirroring observations in the concentration-dependent group.

### 2.7 *In Vivo* Particle Distribution through CED and Tight Junction Disruption

In comparison to conventional drug delivery methods for brain tumor treatment, convection-enhanced delivery (CED) offers several advantages. It circumvents the blood–brain barrier (BBB) and enables the injection of therapeutic agents, whether high or low molecular weight, through bulk interstitial flow.^51^ CED ensures targeted delivery to the region where the catheter is positioned and has the potential for real-time nanoparticle distribution monitoring. Unlike diffusion-restricted delivery, CED employs pressure-driven mechanisms, thereby enhancing the interstitial distribution of drugs. A diverse range of therapeutic candidates, including cytotoxic agents, recombinant toxins, viral vectors, and nanocarriers, have been investigated for feasibility. As an example, paclitaxel (Taxol), an FDA-approved cancer therapeutic drug, has been subjected to experimentation within the context of CED.^52^

**Figure 6:**
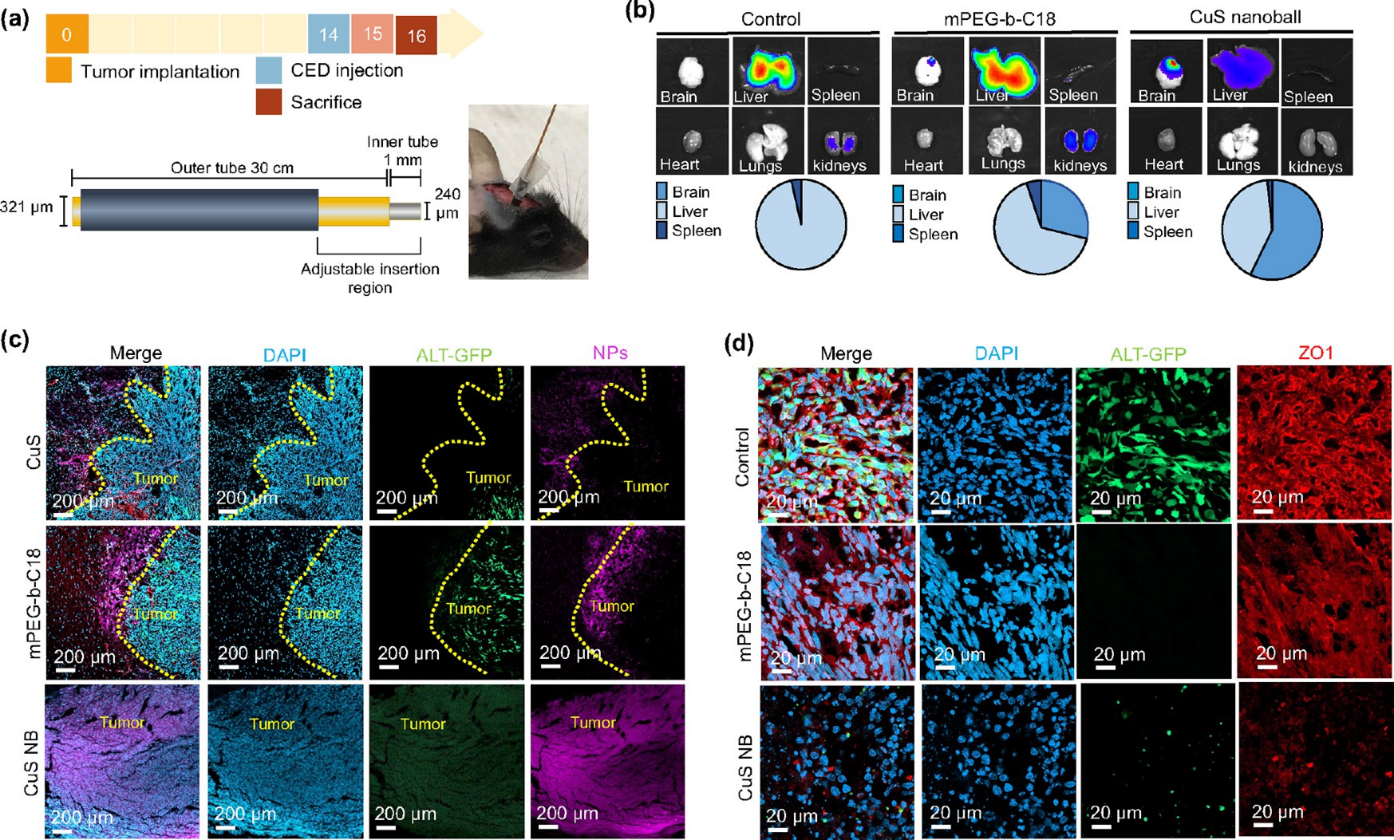
CED and *in vivo* study. (a) CED device design and *in vivo* treatment schedule. (b) *In vivo* IVIS organ biodistribution images of control, mPEG-*b*-C18 micelle-, and CuS NB-treated mice at 48 h post-treatment. (c) CLSM images of brain tumor slices treated by CuS nanoflakes, mPEG-*b*-C18 micelles, and CuS NB. (d) CLSM images of brain slices showing the expression of ZO-1 after various treatments.

To prevent reflux during injection, a reflux-preventing cannula was devised, as illustrated in Figure 6a. This cannula consists of two catheters, one thinner and the other thicker, to impede the backflow of the injection fluid. Another component on the cannula’s periphery controls the catheter insertion depth. Before application in an animal model, a 0.6 wt % agarose gel was utilized to simulate the mouse brain tissue and examine backflow, and distribution patterns at different flow rates were examined. The results in Figure S9 reveal that at flow rates of 0.5 and 1 μL/min, the infusion displayed a spherical pattern that expanded over time. However, at a 2 μL/min flow rate, backflow along the cannula was observed 2 min after injection and intensified over time. Consequently, for *in vitro* and *in vivo* experiments, we plan to inject at a flow rate of 0.5 μL/min.

Through CED, precise injection of both mPEG-*b*-C18 micelles and CuS NB was facilitated into the brain tumor (Figure 6a). The brain tumor mouse model was established. ALTS1C1-GFP cells (2.2 μL) were injected into healthy female C57BL/6 mouse brains intracranially at a concentration of 2 × 10^7^ cells/mL. The mPEG-*b*-C18 micelles were formulated using nanoprecipitation methods. Two days postinjection, an *in vivo* imaging system (IVIS) was employed to assess nanoparticle accumulation and biodistribution. Figure 6b depicts signals from both mPEG-*b*-C18 micelles and CuS NB in the brain, with CuS NB exhibiting a higher intensity. This suggests that the metabolism and loss of mPEG-*b*-C18 micelles occurred more rapidly, possibly due to their smaller sizes, leading to a strong signal of mPEG-*b*-C18 micelles in the liver.

To assess the integrity of the blood–brain barrier (BBB) at the tumor site prior to treatment, a standard method involving the intravenous administration of Evan’s blue dye was employed. Specifically, Evan’s blue dye (2% in normal saline) was administered intravenously (3 mL/kg) following the establishment of brain tumors in mice. After a 30 min interval, the mice were humanely euthanized, and paraformaldehyde (PFA) 4% was perfused intracardially to eliminate any residual dye within the blood vessels. Subsequently, brains were collected for further analysis. Visual inspection of brain images revealed no discernible dye leakage into the brain tissue, indicating BBB integrity (Figure S10a). Additionally, the injection of 10 000 Da fluorescein isothiocyanate (FITC)-dextran (Merck, CAS no. 60842-46-8) was performed to assess dye leakage specifically at the brain tumor site.^53^ As demonstrated by CLSM images (Figure S10b), FITC-dextran is localized within the blood vessels and does not permeate the brain parenchyma. This observation suggests the integrity of the BBB.

Confocal laser scanning microscopy (CLSM) images in Figure 6c illustrate orthotopic brain tumors following treatment with mPEG-*b*-C18 micelles and CuS NB. Sixteen days after brain tumor implantation, the brain tumor area exhibited a higher density of cell nuclei (blue) in the control group with negligible particle signals (red). The heterogeneous structures of the brain tumor contributed to the uneven distribution of particles. At 48 h postinjection of mPEG-*b*-C18 micelles and CuS NB via CED, substantial accumulation and heterogeneous distribution of particles were observed in the brain tumor area. In contrast, CuS NB disassembled and penetrated, showcasing widespread particle distribution throughout the tumor area. Conversely, in regions comprising the normal brain tissue, particle signals were relatively subdued, potentially attributed to the utilization of CED at the brain tumor site. Through this method, the charge conversion of CuS NB is harnessed to augment tumor permeability, enabling deeper penetration into brain tumors by disrupting cell–cell interactions under weakly acidic conditions.

ZO-1, also referred to as tight junction protein-1, is a 220 kD peripheral membrane protein encoded by the TJP1 gene. Predominantly present in epithelial cells, ZO-1 has been identified in gap junctions between astrocytes according to previous research.^54^ To investigate the destruction of solid tumors by CuS NB, brain slices were examined by using ZO-1 staining. In Figure 6d, the control group displayed a complete ZO-1 signal surrounding cells in the dense tumor area. The distinct ZO-1 signal in the solid tumor region, as opposed to the weaker signal in the normal brain region, may be attributed to forming blood vessel epithelial cells and ALTS1C1 tumor cells composing the solid tumor. Similar observations were made in the group injected with CuS nanoflakes, indicating high biocompatibility features. In contrast, the group injected with CuS nanoballs exhibited a significantly weakened ZO-1 signal compared with the control group. This weakening may be attributed to the membrane-disrupting ability of CuS nanoballs, leading to the loosening of the tumor structure.

### 2.8 *In Vivo* Study of Brain Tumor Cell Apoptosis

**Figure 7:**
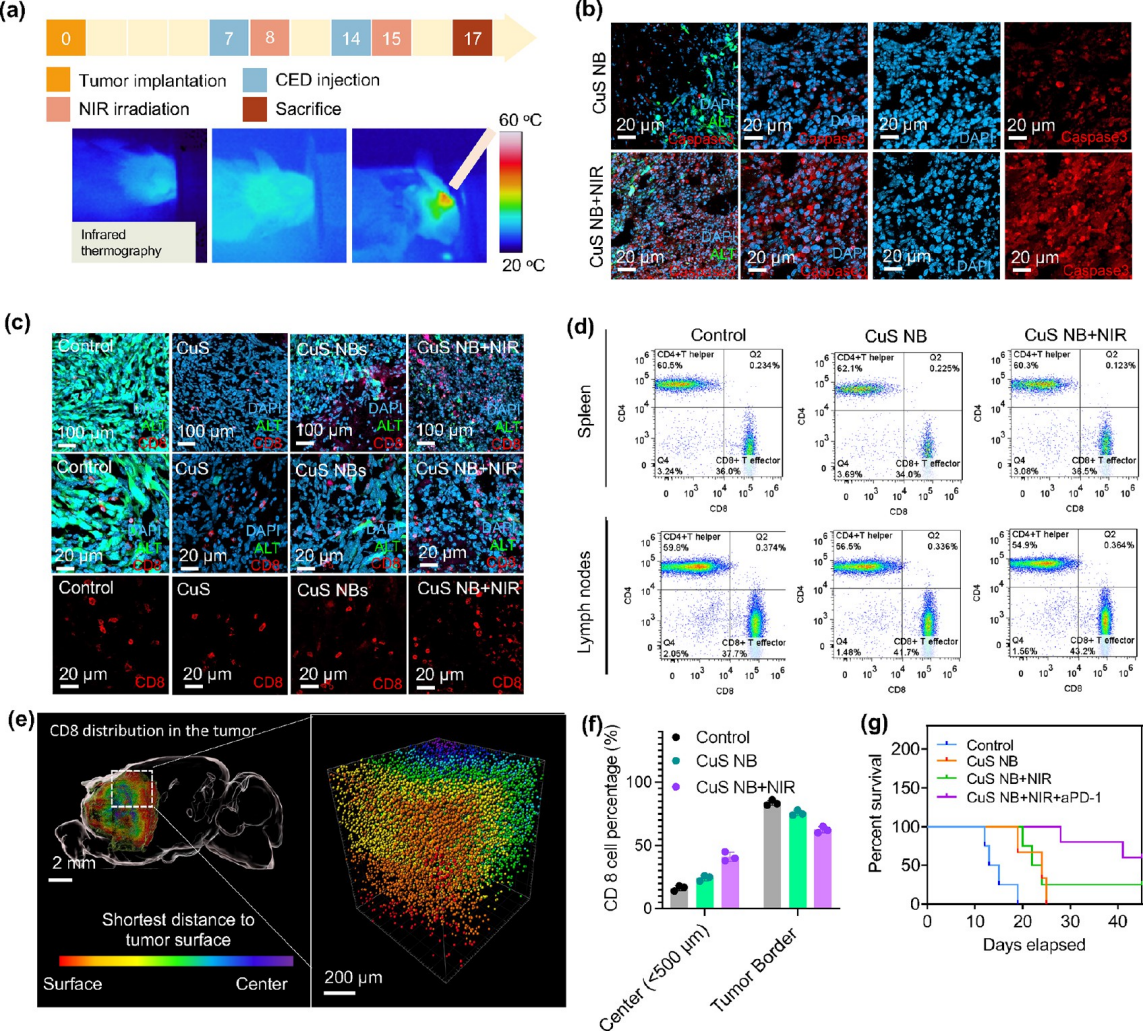
*In vivo* study and immune responses. (a) Treatment schedules and infrared thermal imaging of mice treated with CuS nanoballs via CED and subsequent NIR irradiation on the following day. (b) CLSM images depicting caspase 3 expression in brain tumors following treatment with CuS nanoballs, with and without NIR irradiation. (c) CLSM images of brain slices showing the expression of CD4^+^ and CD8^+^ after various treatments. (d) *In vivo* flow cytometry analysis of the spleen and LN tissue dissected 24 h post-treatment by CuS NB and CuS NB+NIR. (e) Reconstructed 3D images of the entire brains of mice bearing GFP-expressing ALTS1C1 tumors treated with CuS nanoballs and NIR. GFP-expressing ALTS1C1 cells are represented in purple, while CD8-expressing T cells are depicted in white. (f) Quantification of CD8^+^ cells at brain tumors. (g) Survival curves of mice after treatment with control, CuS NB, CuS NB+NIR, and CuS NB+NIR+aPD-1 (*n* = 6).

To assess the therapeutic efficacy of *in vivo* brain tumor treatment, 2.2 μL of GFP-ALTS1C1cells was intracranially injected into the brains of healthy female C57BL/6 mice at a concentration of 2 × 10^7^ cells/mL to establish pre-existing brain tumors. Subsequently, the mice underwent particle treatments on days 7 and 14. To examine photothermal conversion, tumor-bearing mice treated with CuS NB were subjected to 0.8 W cm^–2^ of 1064 nm near-infrared (NIR) irradiation for 2 min, elevating the tumor temperature to 47 °C (Figure 7a), a temperature deemed suitable for thermal treatments. Tumor slices were then analyzed for apoptosis through caspase 3 staining on day 17.

Caspase (cysteine aspartic protease) proteins, with caspase 3 playing an executor role, are involved in apoptosis. In most tumor types, cells evade apoptosis for prolonged survival, accumulating more mutations. In Figure S11, the control group and the CuS nanoflake groups exhibited few caspase 3 signals. Conversely, the group treated with CuS NB showed an increased caspase 3 signal under confocal microscope observation, with a more pronounced increase after 1064 nm NIR irradiation (Figure 7b). A notable contrast was observed: regions without photothermal ablation displayed an intact cell morphology with minimal caspase 3 signal, while other areas exhibited a broken appearance with a heightened caspase 3 signal. These results indicate that the injection of CuS nanoballs induced cellular stress, triggering apoptosis, and that more severe apoptosis occurred after photothermal ablation.

### 2.9 *In Vivo* Study of the T Cell Infiltration and Survival Rate

To understand the infiltration of immune cells, we further analyzed the system by immunofluorescence staining. The tumor slices were stained with anti-CD8, a surface marker of cytotoxic T cells. In Figure 7c, in the control group, some CD8^+^ T cells could be found in the tumor region, indicating that the existence of the tumor would trigger T cell infiltration. In the group with CuS nanoballs, a slightly elevated CD8 signal could be observed, suggesting that tumor destruction caused by the membrane-disrupted ability of CuS nanoballs might facilitate T cell infiltration into the center of the tumor. More infiltrated T cells could be found in the group with NIR irradiation. This might be attributed to generating more tumor antigens by hyperthermia, which further activated more cytotoxic T cells and destroyed tumor tissues, making T cells easier to infiltrate.

To delve deeper into the immune response generated by various treatments, flow cytometry was employed to analyze the number of immune cells in the spleens and lymph nodes, with the gating strategy illustrated in Figure S12. The immune cells in the spleens across different groups exhibited no significant differences; the proportion of CD4^+^ T helper cells remained approximately 60%, and the CD8^+^ cytotoxic T cells hovered around 35%. In contrast, the immune cells from the lymph nodes displayed variations (Figure 7d). Although the percentage of T helper cells decreased with intensified treatment, the percentage of cytotoxic T cells increased from 37.7 to 41.7 and 43.2% following CuS nanoballs and NIR irradiation treatments, aligning with observations under the confocal microscope. The factor contributing to this phenomenon is likely the reduction in tumor size induced by the treatment. To delve deeper into the efficacy of the treatment, comprehensive whole-brain imaging was conducted across the control, CuS nanoball, and photothermal therapy. As illustrated in Figure S13, the signals emanating from brain tumors expressing GFP-ALTS1C1 noticeably diminished following treatment with CuS NB coupled to NIR irradiation. The total tumor size observed corresponded closely with the abundance of T cells present.

We obtained whole-brain images for each treatment group to visualize the distribution of immune cells in the brain. Tumor-bearing mice received CuS nanoballs with or without NIR irradiation after tumor seeding and underwent a two-week treatment. The whole-brain images of the control, CuS nanoball, and photothermal therapy groups are depicted in Figure 7e,f. A noticeable increase in CD8 signal was observed in the treatment groups compared to the control group with all signals concentrated in the tumor area. In the images of the group treated with CuS NB+NIR, CD8^+^ T cells were distributed around the solid tumor area, and the signal of ALTS1C1-GFP highly overlapped with the CD8 signal, indicating an attack by cytotoxic T cells on the tumor cells (Figure 7e). Quantification of CD8^+^ cells distributed within or outside the tumor further revealed that CuS NB+NIR treatment enhanced T cell infiltration into the tumor, suggesting significant induction of T cell infiltration through the combined use of CuS NB and NIR (Figure 7f).

Survival outcomes were monitored for a duration of up to 45 days (Figure 7g). Throughout this observation period, mice underwent various treatments, including PBS (control), CuS NB, CuS NB+NIR, and CuS NB+NIR+aPD-1. The median survival was relatively short in the control groups treated with PBS, approximately 15 days. Conversely, treatment with CuS NB and CuS NB+NIR led to a significant extension in the median survival. Notably, the survival time of mice treated with CuS NB+NIR+aPD-1 was significantly prolonged, indicating an enhancement in T cell infiltration and the efficacy of immunotherapy.

### 2.10 *In Vivo* Study of Antigen Capture and DC Maturation

**Figure 8:**
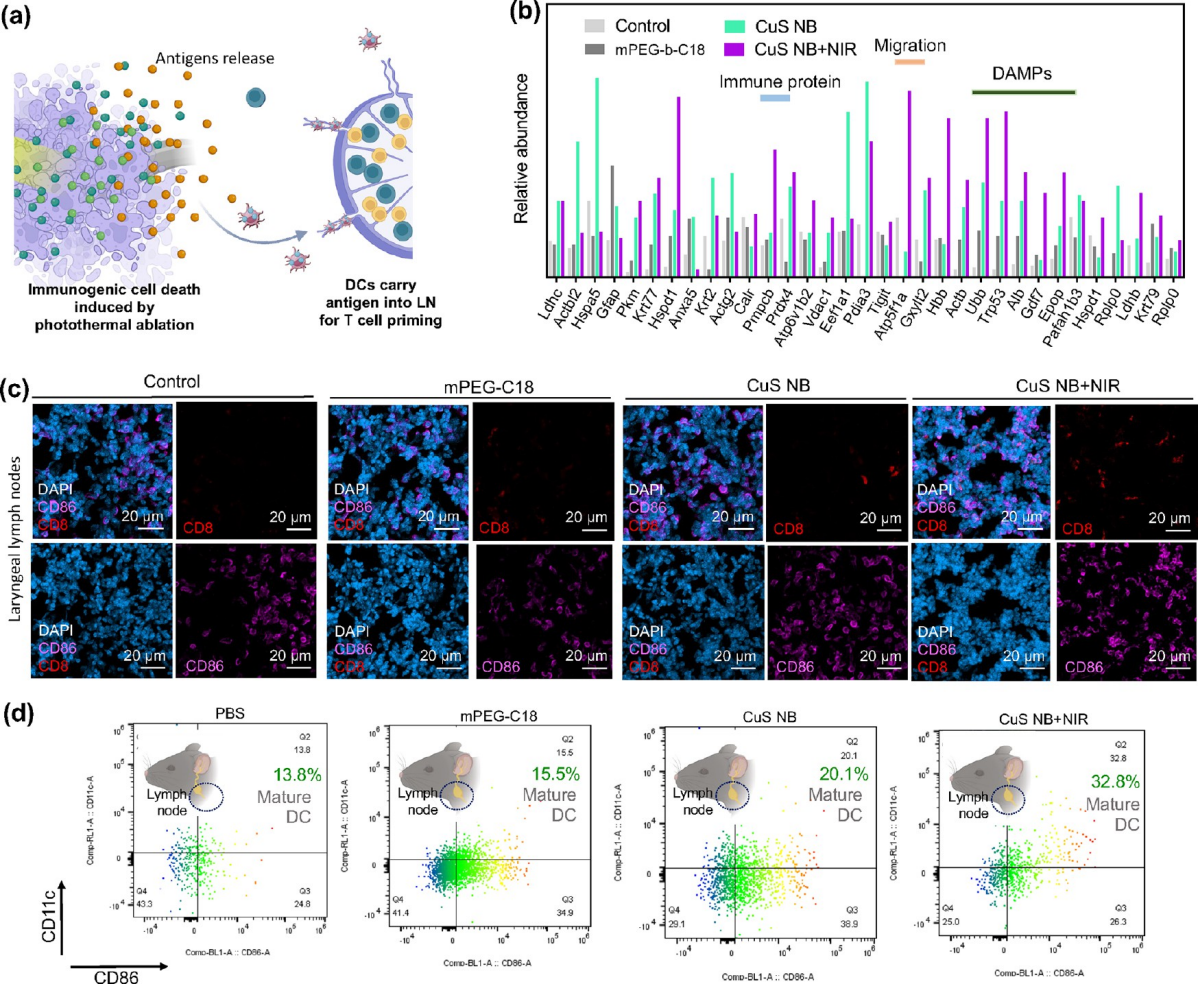
Antigen release and immune responses in laryngeal lymph nodes. (a) Illustration of antigen release and stimulation for immunotherapy. (b) Relative abundance of antigen release after treatment by mPEG-*b*-C18, CuS NB, and CuS NB+NIR. (c) *In vivo* study of mice bearing ALTS1C1 brain tumors treated with mPEG-*b*-C18, CuS NB, and CuS NB+NIR. CLSM images of the LN tissue 24 h postinjection. Blue, purple, and red fluorescence represents nucleus staining with DAPI, DCs with CD86, and T cells with CD8. (d) *In vivo* flow cytometry analysis of the LN tissue dissected 24 h post-treatment by mPEG-*b*-C18, CuS NB, and CuS NB+NIR.

The impact of mPEG-*b*-C18, CuS NB, and CuS NB+NIR on T cell recruitment through antigen capture was evaluated (Figure 8a). The release of neoantigens and damage-associated molecular patterns (DAMPs) by ALTS1C1 cells was examined. The analysis of antigens released and captured by mPEG-*b*-C18, CuS NB, and CuS NB+NIR was conducted using liquid chromatography-mass spectrometry (LC-MS/MS) on an Orbitrap Elite hybrid ion trap-Orbitrap mass spectrometer from Thermo Fisher, USA. In the context of *in vitro* antigen capture, Figure 8b shows the number of different proteins observed on CuS NBs, showing the small effect of minimal surface charge on antigen binding. The results may be attributed to the amphiphilic effect of the molecule.

Among the released antigens, a diverse array of features have been identified. Annexin A5 (Anxa5) is a versatile tool for identifying apoptotic cells and functions as both an immune checkpoint inhibitor and a tumor-homing molecule in cancer treatment. Its involvement extends to various membrane-related processes within exocytotic and endocytotic pathways, with potential roles in cellular signal transduction, inflammation, growth, and differentiation.^55^ Actin beta (ACTB) proteins, highly conserved across species, play crucial roles in cell motility, maintaining cellular structure and integrity and mediating intercellular signaling. Additionally, they provide a structural support to cells. Tumor protein p53 (Trp53) is instrumental in the cellular response to various stresses, preserving genomic integrity and functioning as a critical component of tumor suppression.^56^ The heat shock protein family (Hspd1) contributes to cellular apoptosis and senescence and may facilitate viral attachment and entry into host cells, thereby promoting proliferation and metastasis. Peroxiredoxin 4 (Prdx4) plays a vital role in regulating the redox balance and oxidative folding by reducing H_2_O_2_ levels in the endoplasmic reticulum.^57^ Examining common mechanisms governing protein adsorption on nanomaterials reveals that the nanoparticle’s surface significantly influences protein adsorption. This process involves proteins binding to the particle surface, and the polymer charges influence the adhesion strength of mPEG-*b*-C18, CuS NB, and CuS NB+NIR.

The lymphatic system, including laryngeal lymphatics, is pivotal in orchestrating immune responses throughout the body. Immune cells and molecules transported via the lymphatic system indirectly influence the immune reactions within the brain. This becomes particularly pertinent in conditions where immune responses within the brain are crucial, such as in cases of neuroinflammation or brain tumors.^58^ Lymphatic vessels facilitate the transport of immune cells from peripheral tissues to lymph nodes and back into the bloodstream. In brain immunotherapy, a comprehensive understanding of immune cell trafficking is imperative. This understanding holds significance, as it can profoundly impact the efficacy of therapies designed to modulate immune responses within the brain.

The impact of CuS NB on the *in vivo* recruitment of dendritic cells (DCs) was investigated in mice bearing ALTS1C1. Twenty-four h postinjection, lymph nodes were dissected, and the quantification of DCs and T cells in the laryngeal lymph was examined (Figure 8c). Confocal laser scanning microscopy (CLSM) images of the lymph node tissue stained with CD86 (a typical marker for the upregulation of DCs) and CD8^+^ (a characteristic marker for the upregulation of the T cell surface, indicating immune activity) were presented. The fluorescence colors blue, green, and purple, respectively, represented nucleus staining with DAPI, DCs with CD86, and T cells with CD8^+^. The results revealed four distinct groups in the lymph nodes, exhibiting a more robust expression of CD86, indicative of the accumulation of capturing nanoparticles in the lymph nodes. Notably, the CuS NB+NIR group displayed a 3-fold greater expression of CD8^+^ compared to the other groups, suggesting enhanced immunotherapy. This heightened CD8^+^ expression may contribute to the increased efficacy of immunotherapy in this specific group. A noticeable enhancement in DC maturation was observed through flow cytometry analysis following treatment with CuS NB combined with NIR irradiation (Figure 8d).

The concentrations of immune factors, such as tumor necrosis factor-α (TNF-α), interferon-γ (IFN-γ), interleukin-10 (IL-10), and interleukin-12 (IL-12) in brain tissues treated with various samples, were quantified using ELISA kits. As presented in Figure S14, the results illustrated enhanced levels of IFN-γ, IL-10, and IL-12 in groups treated with CuS NB and CuS NB+NIR compared to the control group. This elevation in the immune factors suggests the induction of an immune response. Additionally, Figure S15 delineates the impact of CuS NB and CuS NB+NIR treatments on liver and kidney functions. The results indicated subtle treatment effects on these organs, implying a harmonious interaction of the tumor-targeted therapeutic agents with minimal adverse effects on liver and kidney functions.

To further assess the biosafety of the nanoplatform and its neural impact and influence on animal behavior, we investigated animals subjected to various treatments. In Figure S16a,b, we scrutinized the regenerated neurofilament cells utilizing the axonal marker Neurofilament 200 (NF200). The CuS NB+NIR+aPD-1 group exhibited significantly heightened expression of the NF200 marker compared with other groups. This finding suggests that the administered treatment, alongside tumor inhibition, also facilitated nerve cell growth.

To further gauge the efficacy of promoting recovery and restoring brain function postoperative brain tumor treatment, we conducted animal behavior tests subsequent to surgical procedures and treatment (Figure S16c). Specifically, female C57BL/6 mice, aged 7 weeks, were divided into four distinct groups, each consisting of five individuals: CuS NB, CuS NB+NIR, and CuS NB+NIR+aPD-1. Over a 42-day period, we conducted weekly animal behavior experiments to evaluate the lasting effects of the treatments. To assess forelimb movement, we employed the cylinder test (Figure S16c). Since the postoperative brain tumors were applied to the left brain, mice typically favored using their left limb for exploring their surroundings, leading to a decrease in the use of the right limb (indicated as positive) and an increase in the use of the left limb (indicated as negative). The cylinder test revealed a progressive decline in performance for the untreated group. In contrast, the CuS NB+NIR+aPD-1 groups showed a tendency for forelimb use to approach that of both limbs, indicating a more balanced recovery. Moreover, in the grid test, the untreated group displayed the highest number of foot faults, while the CuS NB+NIR+aPD-1 group exhibited fewer foot faults than the other groups, suggesting an improved hind-limb function in this group. These results underscore the potential biosafety of this platform.

For the laser penetration issue, we held close consultations with Prof. Yu-Ren Lu (MD, PhD), a distinguished neurosurgeon specializing in brain tumors in Chang Gung Memorial Hospital in Taiwan with over 20 years of experience. Prof. Lu indicated the potential clinical usage of the delivery system and NIR II laser irradiation in brain tumor treatment. Currently, laser interstitial thermal therapy (LITT) stands out as a minimally invasive method, delivering precise and controlled laser energy directly into tumor tissue in clinics.^59−63^ This approach offers significant advantages, including smaller incisions, minimal tissue damage, and faster patient recovery compared with conventional open surgery. Despite LITT’s versatility, careful patient selection is paramount, considering tumor characteristics and proximity to critical structures. Furthermore, the tumor cells infiltrated within normal tissues may evade the effects of LITT. Our integrated system, coupled with immunotherapy and NIR II technology, has demonstrated encouraging outcomes in treating brain tumors. By facilitating precise delivery and augmenting the immune response, our approach holds significant promise for improving therapeutic efficacy in this complex clinical domain.

## 3 Conclusions

In summary, we developed a membrane-disrupted nanodevice comprising a pH-responsive polymer and CuS nanoflakes, serving as a cellular leakage and NIR II photoconversion agent to enhance tumor permeability and T cell infiltration. Administered through convection-enhanced delivery, these particles effectively bypass the blood–brain barrier, augment tumor permeability, and localize within deep brain tumors. The polymer reduces cell–cell interactions at the tumor site and induces cellular leakage. Upon exposure to low-power NIR II irradiation, CuS generates heat, facilitating the thermolytic penetration of nanoflakes into the tumor. This process induces cell death and subsequent antigen release. The positively charged polymer functions as an antigen depot, capturing autologous tumor-associated antigens and presenting them to dendritic cells, thereby ensuring sustained immune stimulation. This self-cascading penetrative immunotherapy not only amplifies the immune response to postoperative brain tumors but also holds promise for broader applications in enhancing immune responses to various types of tumors.

## 4 Experimental Section

### 4.1 Synthesis of CuS Nanoflakes

Briefly, the synthesis process involved introducing 0.5 mmol of CuCl, 6 mmol of oleylamine, 1 mmol of oleic acid, and 10 mL of octadecene into a three-neck flask followed by degassing at 100 °C for 20 min.^45^ Subsequently, the solution underwent nitrogen purging and was heated to 180 °C. During this phase, CuCl formed coordination bonds with oleylamine and oleic acid during this phase through their amino and C=C double bond, respectively. Upon reaching a stable temperature, a rapid injection of *S*-oleylamine solution, prepared by dissolving 2 mmol of S in 2 mL of oleylamine under nitrogen flow, was carried out. The reaction proceeded for 10 min before being halted through cooling. The resulting CuS nanoflakes underwent purification via three rounds of centrifugation with a methanol and hexane mixture (3:1 ratio) and were collected as a residue after drying.

### 4.2 Synthesis of CuS NB

The membrane-disrupting/pH-responsive amphiphilic polymer, methoxy poly(ethylene glycol)-benzoic-imine-octadecane (mPEG-*b*-C18), was generously provided by the Wen-Hsuan Chiang lab at National Chung Hsing University. The synthesis process involved two key steps. First, methoxy poly(ethylene glycol) benzaldehyde (mPEG-CHO) was synthesized by esterifying mPEG and *p*-formylbenzoic acid using *N*,*N*′-dicyclohexylcarbodiimide (DCC)/4-dimethylaminopyridine (DMAP). Second, mPEG-*b*-C18 was synthesized by the Schiff base reaction. To elaborate, mPEG-CHO (500 mg, 1 equiv), 1-octadecanamine (80 mg, 1.2 equiv), and DMAP (61 mg, 2 equiv) were dissolved in dichloromethane (5.0 mL) and stirred for 36 h at room temperature (Figure 1). After dichloromethane was removed under reduced pressure, the resulting product was dissolved in DMSO and dialyzed (Cellu Sep MWCO 1000) against pH 8.0 deionized water at 4 °C to eliminate DMSO. Ultimately, mPEG-*b*-C18 was obtained through freeze-drying.

### 4.3 Characterizations

The morphologies of CuS nanoflakes and CuS nanoballs were examined by using field-emission scanning electron microscopy (FE-SEM, JSM-7000F, Japan) and TEM (JEM-2100, Japan). For TEM analysis, the nanoparticles were desiccated on a copper grid. The elemental mapping of TEM (JEM-2100, Japan) was carried out by using electron energy loss spectroscopy (EELS) spectra. The nanoparticles' zeta potential and hydrodynamic radius of the nanoparticles were assessed via dynamic light scattering (DLS, Nano-ZS, Malvern, UK). In this process, samples were appropriately diluted with ddH_2_O in a plastic cuvette, and the nanoparticle distribution was measured by observing the interaction of light with small particles over a specified duration. The X-ray powder diffractometer (X-RPD) was employed to ascertain the solid crystal structure and lattice of the CuS nanoparticles. The composition of the nanoparticles was investigated by using a thermogravimetric analyzer (TGA), with the samples undergoing overnight drying. X-ray spectroscopy (XPS, PHI Quantera SXM, Japan) was conducted to identify the elements and composition of the nanoparticles.

### 4.4 Cell Culture

Murine astrocytoma cells (ALTS1C1) were cultured in Dulbecco’s modified Eagle’s medium (DMEM) supplemented with 1% penicillin–streptomycin (PS) and 10% fetal bovine serum (FBS) in a 10 cm dish plate at 37 °C in a 5% CO_2_ incubator. The culture medium was diligently refreshed every 2 days. Subculturing involved aspirating the spent DMEM, gently washing the cells with PBS, and then introducing warm 0.25% trypsin-EDTA into the dish. The mixture was incubated at 37 °C for 3 min followed by the addition of 2 mL of a medium to halt the trypsin reaction. Cell counting and viability assessment were performed using a cell counter and trypan blue.

For thawing frozen cells, the cryopreserved cells were immediately placed in a 37 °C water bath upon retrieval from the cell bank. Subsequently, the cells were gently introduced into a medium-filled dish. The following day, the medium was replaced to eliminate the cryoprotectant (7% DMSO), and a fresh medium was added every 2 days for continuous cell culture.

### 4.5 Cellular Uptake, Distribution, and Cytotoxicity

The cytotoxicity of ALTS1C1 cells under various treatments was assessed using the PrestoBlue cell viability reagent. Initially, ALTS1C1 cells were seeded into a 96-well plate at a density of 10^4^ cells per well and cultured for 24 h at 37 °C. Subsequently, the culture medium was replaced with a fresh medium containing different concentrations of CuS, mPEG-*b*-C18, and CuS NB and cultured for 24 h. Following this incubation, 10 μL of PrestoBlue was added to each well and incubated at 37 °C for 10 min. Absorbance values were measured using an ELISA reader at an excitation wavelength of 530 nm and an emission wavelength of 590 nm. The untreated group served as the control, normalized to 100% with standard deviation, while the cell viability of the treated groups was determined based on emission values.

The nanoparticles were labeled with a fluorescent dye to assess the cellular uptake of CuS nanoflakes and CuS NB (Cy5.5 Amidite). Cy5.5 was dissolved in DMSO and added directly to an Eppendorf containing CuS nanoflakes followed by sonication. After sonication, excess dye was eliminated through ethanol washes and centrifugation (1–3 times) followed by drying. Stained CuS nanoflakes were then dispersed in the medium for further experiments. For CuS nanoballs, stained CuS nanoflakes were encapsulated with mPEG-*b*-C18 using the previously mentioned protocol.

ALTS1C1 cells were seeded in a confocal plate at 10^5^ cells per dish for 24 h in 1 mL of DMEM containing 10% FBS and 1% PS. The following day, the old medium was replaced, and a fresh medium with varying concentrations of Cy5.5-labeled nanoparticles was added to each plate followed by incubation at 37 °C for different time points. Afterward, the medium was removed, and the cells were washed with PBS twice. Cells were fixed with 3.7% formaldehyde for 15 min, permeabilized with 0.1% Triton X-100 for 15 min, stained with 1 μg/mL 4′-6-diamidino-2-phenylindole (DAPI) for 15 min for cell nuclei, and stained with Alexa Fluor 488 phalloidin (F-actin, 300 units/mL) for 2 h to visualize the cytoskeleton. Washes with PBS were performed between each step. Finally, cells were mounted with an antifade fluorescence mounting medium and observed under a confocal laser scanning microscope (LSM-800).

### 4.6 Membrane Disruption Test

The membrane leakage test, primarily modeled after that of Kuroda et al. with certain modifications, was conducted as follows.^50^ Initially, ALTS1C1 cells were seeded in a 6-well plate at a density of 5 × 10^4^ cells for 24 h at 37 °C. The medium was then replaced with a fresh medium containing varying concentrations of mPEG-*b*-C18 or CuS NB concentrations and cultured for 2 h at 37 °C. Following the 2 h incubation, the medium containing the materials was removed, and the cells were gently washed with PBS 1–2 times to eliminate excess particles. DAPI was added and incubated for 30 min to allow the penetration to the cells followed by a gentle wash with PBS. Subsequently, a LIVE/DEAD viability/cytotoxicity kit was employed, and the cells were observed under a confocal microscope.

For SEM sample preparation, ALTS1C1 cells were seeded in a 6-well plate with 10^5^ cells per well for 24 h at 37 °C. The medium was then replaced with 100 μg/mL CuS nanoballs and incubated at 37 °C. Following incubation for 1, 2, 3, and 4 h, the cells were washed with PBS and treated with trypsin to collect the cell suspension. The cell suspension was fixed with 2% paraformaldehyde and 2.5% glutaraldehyde in a 0.2 M pH 7.4 cacodylate buffer at 4 °C overnight. After fixation, the cell suspension was washed with a cacodylate buffer, dehydrated with graded ethanol at 4 °C, and treated with hexamethyldisilane (HDMS) for 30 min to dry the samples chemically. The samples were then dropped onto poly-l-lysine-coated coverslips and examined using SEM.

### 4.7 *In Vitro* Antitumor Activity

For fluorescence image observation, ALTS1C1 cells were seeded in a 6-well plate at a density of 10^5^ cells per well and incubated for 24 h at 37 °C. The medium was then replaced with a fresh medium containing varying concentrations of CuS NB and cultured for 2 h at 37 °C. After the 2 h incubation, the cells were exposed to a 1064 nm NIR laser (0.8 W/cm^2^) for 5 min or left untreated followed by an additional 6 h incubation. Subsequently, the medium was removed, and the cells were gently washed with PBS. The cells were stained with a LIVE/DEAD viability/cytotoxicity kit and observed under a confocal microscope.

### 4.8 Statistics and Reproducibility

Statistical analyses were conducted using GraphPad Prism software (version 10.0) based on data obtained from three or more independent experiments. Error bars in the figures represent the standard deviation (SD) derived from three or more independent experiments. To evaluate differences between groups, a one-way analysis of variance (ANOVA) was initially performed to evaluate differences between groups followed by either Dunnett’s or Tukey’s multiple-comparison tests, as specified in the figure legends. *P < 0.05, **P < 0.01, ***P < 0.001, and ****P < 0.0001 were considered statistically significant.

## Supporting Information Available

Table of the proportion of each preparation method alongside its corresponding appearance stability (Table S1); weight percentages of CuS, CuS nanoballs, and mPEG-*b*-C18 at both 101 and 800 °C (Table S2); X-ray photoelectron spectroscopy (XPS) outcomes for CuS nanoballs at pH 5.8 and 7, focusing on Cu 2p and S 2p analysis (Figure S1); schematic illustration of hydrolysis of the benzoic-imine linker form mPEG-*b*-C_18_ (Figure S2); DLS measurements of CuS NB following irradiation with 1064 nm near-infrared (NIR) light for 1 min and conducted at various pH values (Figure S3); CLSM images capturing the progression of ALTS1C1-GFP tumor spheroids cocultured with 20 μg/mL CuS nanoballs over time (Figure S4); CLSM images of the viability of ALTS1C1 tumor spheroids following different treatments (Figure S5); TEER measurements of bEnd.3 cells and human umbilical vein endothelial cells after treatment by mPEG-*b*-C18 and CuS NB in a Transwell at 24 h postinjection (Figure S6); concentration-dependent cellular uptake of CuS nanoflakes (Figure S7); time-dependent cellular uptake of CuS nanoballs (Figure S8); evaluation of flow rates in 0.6 wt % agarose gel using trypan blue as an indicator (Figure S9); brain image after Evan’s blue dye was administered intravenously following the establishment of brain tumors in mice (Figure S10); CLSM images depicting tumor slices subjected to various treatments (Figure S11); gating strategy of flow cytometry (Figure S12); whole-brain images of GFP-ALTS1C1 brain tumor mice treated with CuS NB and CuS NB+NIR (Figure S13); concentrations of immune factors such as tumor necrosis factor-α (TNF-α), interferon-γ (IFN-γ), interleukin-10 (IL-10), and interleukin-12 (IL-12) in brain tissues treated with various samples quantified using ELISA kits (Figure S14); biochemical indices of the liver and kidney after 72 h of treatment (Figure S15); *in vivo* neural recovery and animal behavior in brain tumors treated with CuS NB, CuS NB+NIR, and CuS NB+NIR+aPD-1 (Figure S16) (PDF)
