# Supplementary material for: A Self-Cascade Penetrating Brain Tumor Immunotherapy Mediated by Near-Infrared II Cell Membrane-Disrupting Nanoflakes via Detained Dendritic Cells
Source: ACS Nano. 2024 Jul 2;18(28):18712–28. doi: 10.1021/acsnano.4c06183 (PMC11256899; doi:10.1021/acsnano.4c06183)
Supplement: Supplementary file 1 — nn4c06183_si_001.pdf [file nn4c06183_si_001.pdf]

Supporting Information

A Self-Cascade Penetrating Brain Tumor Immunotherapy-Mediated by Near-Infrared-II Cell Membrane-Disrupting Nanoflakes *via* Detained Dendritic Cells

Bhanu Nirosha Yalamandala[1], *Yu-Jen Chen*[1], *Ya-Hui Lin*[1,2], *Thi My Hue Huynh*[1], *Wen-Hsuan Chiang*[3], *Tsu-Chin Chou*[4], *Heng-Wei Liu*[5,6,7], *Chieh-Cheng Huang*[8], *Yu-Jen Lu*[9,10], Chi-Shiun Chiang[1], Li-An Chu[1,2], *Shang-Hsiu Hu*[1,4,]*

[1] Department of Biomedical Engineering and Environmental Sciences, National Tsing Hua University, Hsinchu 300044, Taiwan
[2] Brain Research Center, National Tsing Hua University, Hsinchu, 300044 Taiwan
[3] Department of Chemical Engineering, National Chung Hsing University, Taichung 402, Taiwan
[4] Institute of Analytical and Environmental Sciences, National Tsing Hua University, Hsinchu, 300044 Taiwan
[5] Department of Neurosurgery, Shuang Ho Hospital, Taipei Medical University, New Taipei City, 23561, Taiwan
[6] Taipei Neuroscience Institute, Taipei Medical University, Taipei, 11031, Taiwan
[7] Department of Surgery, School of Medicine, College of Medicine, Taipei Medical University, Taipei, 11031, Taiwan
[8] Institute of Biomedical Engineering, National Tsing Hua University, Hsinchu, 300044 Taiwan
[9] Department of Neurosurgery, Chang Gung Memorial Hospital, College of Medicine Chang Gung University, Taoyuan, 33305, Taiwan
[10] College of Medicine, Chang Gung University, Kwei-San, Taoyuan, 33302, Taiwan

E-mail addresses: shhu@mx.nthu.edu.tw

EXPERIMENTAL SECTION

Penetration of the Particles in ALTS1C1 Spheroids. A microfluidic chip was employed in the formation of ALTS1C1 spheroids and subsequent experiments. Prior to cell experiments, the microfluidic chips were filled with PBS to eliminate bubbles and minimize device toxicity. Subsequently, the degassed chips were exposed to UV light for overnight sterilization. Once prepared, the remaining PBS in the chip was removed, leaving a minimal amount, and 500 µL of medium was added. ALTS1C1 cells were harvested using 0.05% trypsin, concentrated or diluted to a concentration of 10^6 cells per mL, and 300 µL of the cell suspension was directly loaded into the chip. During this step, cells precipitated into the micro-wells through gravitational force. The device was then placed

in a 37°C incubator with 5% CO_2 for 24 hours. Following cell loading, the cells aggregated into 3D structures within each micro-well. This microfluidic chip was utilized as an in vitro 3D model to observe cellular uptake and nanoparticle penetration.

For the analysis of cellular uptake and cell morphologies of nanoparticles in tumor spheroids, 1 mL of nanoparticle-containing medium was gently added to the microfluidic chip. Black controls were conducted using tumor spheroids without treatment. After loading the nanoparticles, the chip was incubated at 37 ℃ for 24 hours. Subsequently, the tumor spheroids were fixed with 4% formaldehyde for 1 hour. Following fixation, cell nuclei and cytoskeleton were stained with DAPI and F-actin, respectively. For additional ZO-1 staining, cells were incubated with PBS containing 10% goat serum, 0.3M glycine, 1% BSA, and 0.1% tween for 1 hour at room temperature to permeabilize cells and block non-specific binding after formaldehyde fixation. The Anti-ZO1 tight junction protein antibody was added and incubated at 4 ℃ overnight. The secondary antibody was then added for 1 hour at room temperature. The outcomes were observed using an optical microscope and a confocal microscope.

Transendothelial electrical resistance (TEER) measurement. TEER measurement is a noninvasive technique for indirectly evaluating the tight junction integrity of the cells through the measurement of the electrical resistance across a cellular layer. The evaluation of TEER value was performed by using a commercially available TEER measurement equipment (EVOM2, World Precision Instruments) with a chopstick electrode pair (STX3, World Precision Instruments, USA). The TEER values of Transwell membrane were measured in triplicate. The TEER values were finally determined as follows:

$$\mathrm{TEER}(\Omega\cdot\mathrm{cm}^2)=(R_T(\Omega)-R_B(\Omega))\times A(\mathrm{cm}^2)$$

where R_T is the total resistance across the cellular monolayer on the membrane, R_B is the blank resistance of the membrane only (without cells).

***In Vivo* Experiments.** Female C57BL/6 mice aged 6-7 weeks (procured from the National Laboratory Animal Center, NLAC, Taiwan) served as the animal model for distribution and anti-tumor activity

experiments. To establish a brain cancer-bearing animal model, 2.2 μL of ALTS1C1 brain cancer cells were intracranially injected at a concentration of 2×10^7 cells per ml. To track the accumulation of nanoparticles, all nanoparticles were labeled with Cy5.5 and administered via Convection Enhanced Delivery (CED) with a total volume of 5 μL and a flow rate of 0.5 μL/mL.

For immunofluorescence staining, brains were fixed with 4% paraformaldehyde (PFA) overnight, dehydrated in 30% (w/v) sucrose solution for 24-48 hours, and then frozen in O.C.T before sectioning into 10 μm thickness slices. The slices underwent methanol immersion at -20°C for 5-10 minutes, followed by three washes with PBS. A blocking buffer (5% BSA) was applied to the tissue at room temperature for 1 hour to reduce non-specific binding of antibodies. Subsequently, the samples were stained with rabbit anti-CD8, rat anti-CD31 primary antibodies to visualize T cells and blood vessels at 4°C for 12 hours. Anti-ZO-1 and cleaved caspase-3 (Asp175) antibody were also utilized to assess the membrane lysis effect.

Additionally, the IVIS spectrum (*In vivo* Imaging System 200 series, Caliper LifeScience, USA) was employed to examine the tumor site accumulation of nanoparticles labeled with Cy5.5 fluorescence (excitation: 640 nm, emission: 700 nm). In vivo targeting for photothermal therapy and chemotherapy involved nanoparticle injection on days 10 and 13 after tumor seeding (day 0), followed by irradiation with 1064 nm near-infrared (NIR, 0.8 W/cm^2) for 1 minute on the subsequent day. Anti-PD1 was administered through tail vein injection once a day on days 11 and 13. All measurements were conducted in triplicate.

For *in vivo* flow cytometry, spleen and lymph nodes from brain tumor mice models were collected following nanoparticle injection and NIR irradiation. The harvested organs were dissected into small pieces in Hanks' Balanced Salt Solution (HBSS) and RBC lysis buffer. Collagenase, D-NAse, and dispase were added to isolate single cells, and the tissue solution was shaken at 190 rpm for 60 minutes. A 70 μm filter was then used to remove residue, and the solution was centrifuged at 800 g for 5 minutes to collect the cells. The cell precipitate was resuspended in HBSS, and a blocking buffer containing

goat serum and FC blocker purified rat anti-mouse CD16/CD32 was added to prevent nonspecific binding of antibodies.

Anti-CD45, anti-CD3ε, anti-CD4, and anti-CD8a were added for staining, and the cells were incubated for 1 hour at 4 °C to identify immune cells. After staining, the cells were collected by centrifugation and resuspended in PBS. Subsequently, the samples were examined using the Attune NxT acoustic focusing cytometer.

Method	mPEG-b-C_{18}	CuS	PVA	Appearance	Sample
Dialysis	1 mg/ml	0.5 mg/ml	-	×	A
	1.5 mg/ml			×	B
	1.95 mg/ml			×	C
	1 mg/ml	2 mg/ml	-	×	D
	2 mg/ml			×	E
	4 mg/ml			×	F
Emulsion solvent evaporation	4 mg/ml	4 mg/ml	0.5 wt%	○	G
	6 mg/ml			○	H
	8 mg/ml			○	I
	4 mg/ml	4 mg/ml	0.1 wt%	×	J
	-	4 mg/ml	0.5 wt%	○	K

Table S1. Table of detail proportion of the proportion of each preparation method alongside their corresponding appearance stability. In the table, utilize "O" to signify favorable dispersity and "X" to indicate inadequate dispersity.

	Weight % at 100℃	Weight % at 800℃	Weight loss
Cus	98.92	77.23	21.69
Cus nanoball	97.35	31.67	65.68
mPEG-b-C$_{18}$	98.27	4.92	93.35

Table S2. The weight percentages of CuS, CuS nanoballs, and mPEG-b-C18 at both 101°C and 800°C. The weight loss percentages were employed to calculate the loading capacity of CuS.

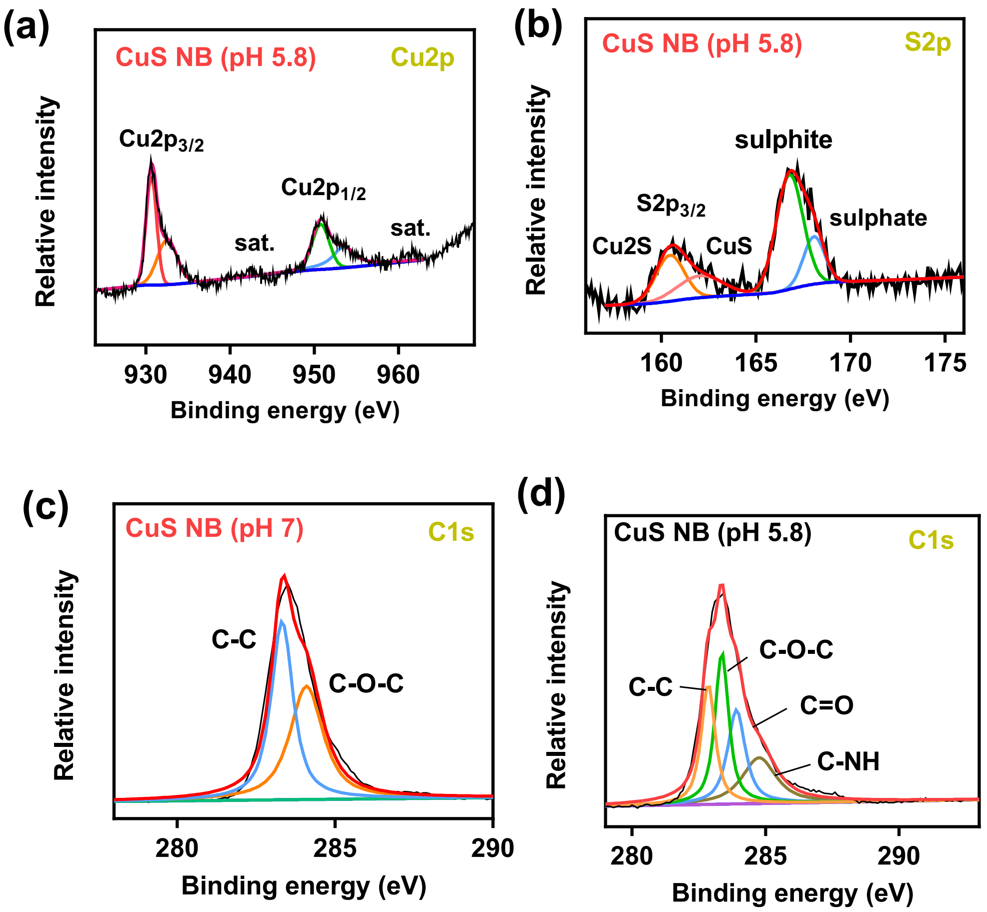

Figure S1. X-ray Photoelectron Spectroscopy (XPS) outcomes for (a) CuS nanoballs at pH 5.8, focusing on Cu2p; (b) S2p analysis. XPS spectra depicting (c) C1s analysis and (d) N1s analysis for CuS nanoballs at pH 7.

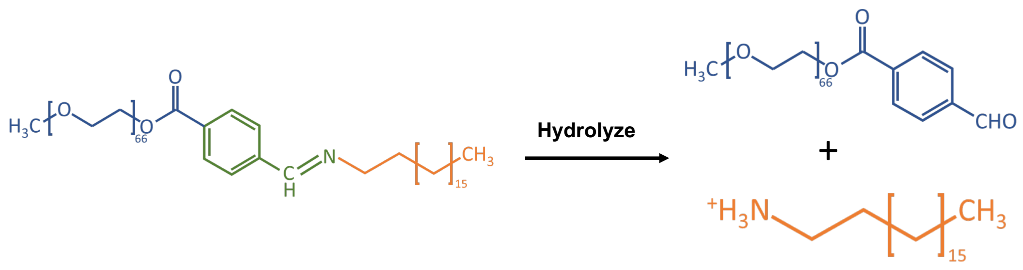

Figure S2. Schematic illustration of hydrolysis of benzoic imine linker form mPEG-b-C_{18}.

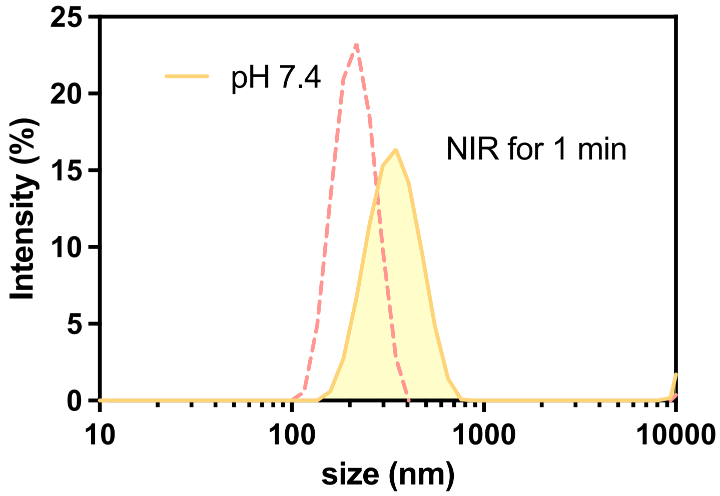

Figure S3. DLS measurements of CuS NB following irradiation with 1064 nm Near-Infrared (NIR) light for 1 minute, and conducted at various pH values.

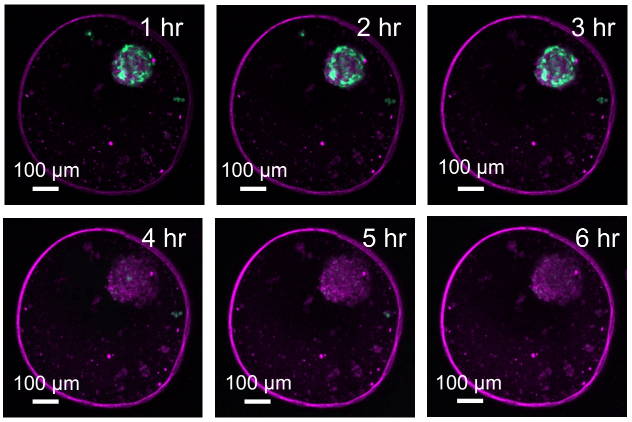

Figure S4. CLSM images capturing the progression of ALTS1C1-GFP tumor spheroids co-cultured with 20 μg/mL CuS nanoballs over time. The CuS nanoballs, labeled with Cy5.5, are visualized in violet, while the ALTS1C1-GFP is presented in green.

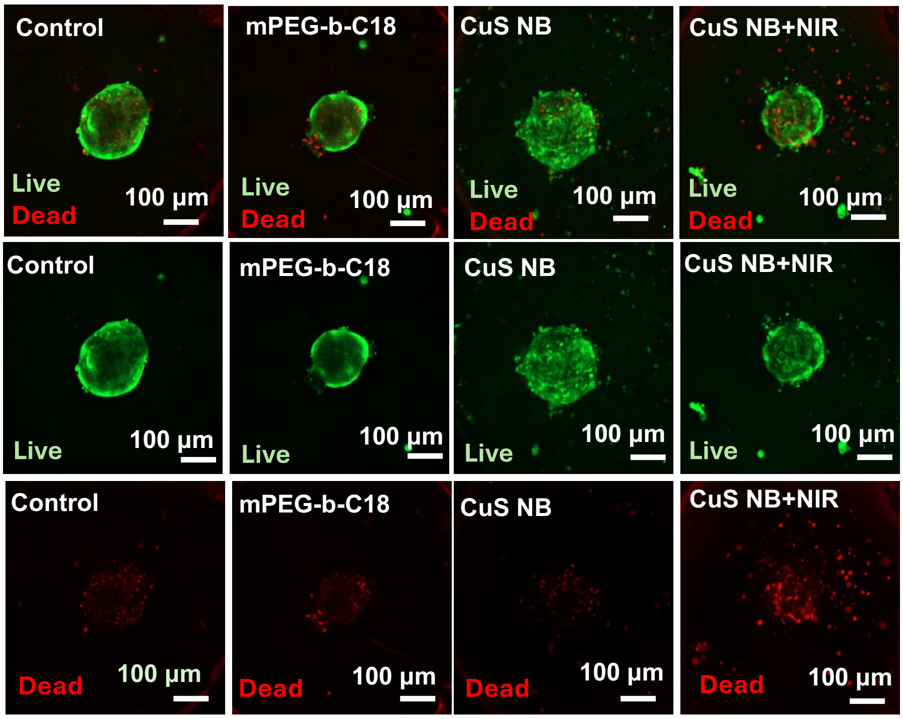

Figure S5. CLSM images of the viability of ALTS1C1 tumor spheroids following different treatments, as assessed by live/dead cell staining.

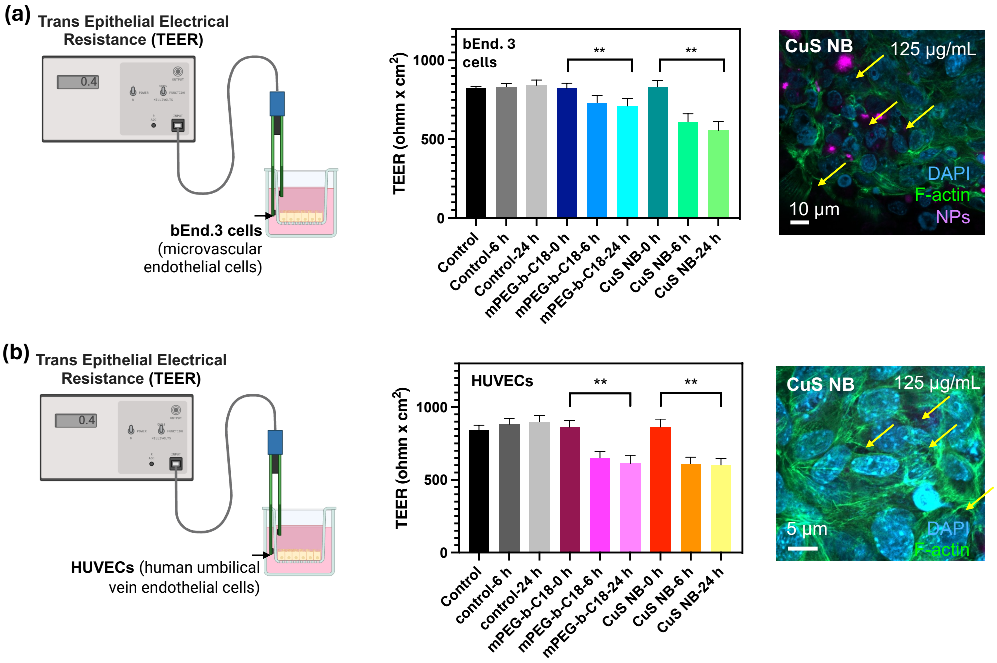

Figure S6. TEER measurements of (a) bEnd.3 cells and (b) human umbilical vein endothelial cells (HUVECs) after treated by mPEG-b-C18 and CuS NB in a Trenawell at 24 h postinjection (n = 6). The bEnd.3 cells, comprising mouse cerebral microvascular endothelial cells, was seeded onto a Trenawell substrate. CLSM image of bEnd. 3 cells or HUVECs in a Trenawell of CuS NB for 24 h.

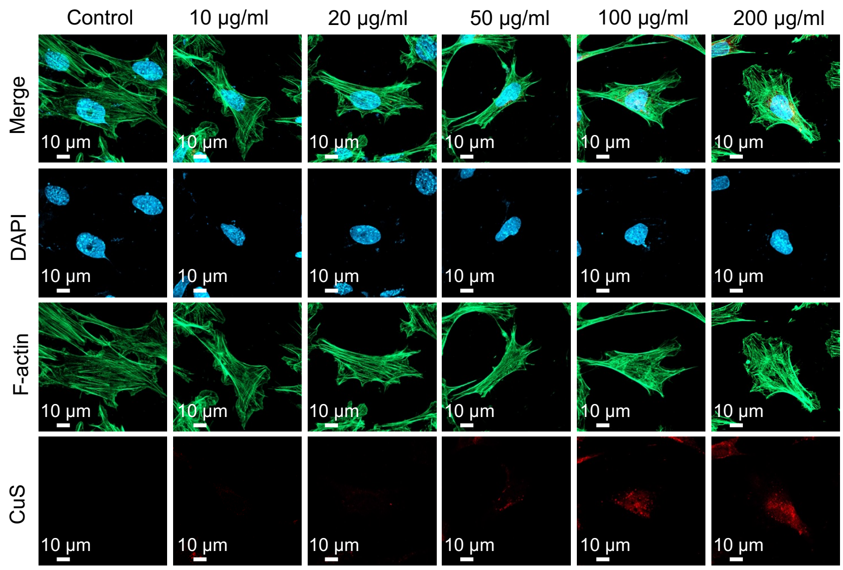

Figure S7. Concentration-dependent cellular uptake of CuS nanoflakes. The cell nucleus was stained with DAPI, appearing as blue; the cytoskeleton was labeled with F-actin, represented in green; and the CuS nanoflakes were stained with RITC, presented in red.

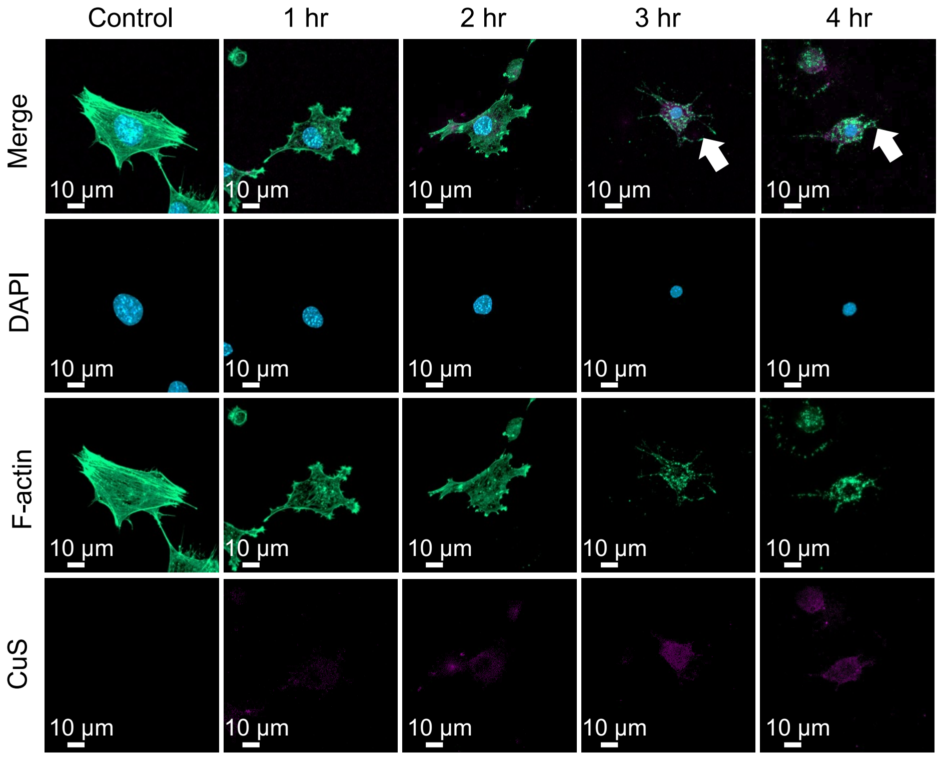

Figure S8. Time-dependent cellular uptake of CuS nanoballs. Cell nucleus was stained with DAPI and presented as blue; cytoskeleton was stained with F-actin and presented as green; CuS nanoballs were stained with Cy5.5 and presented as violet. Cell debris was indicated by white arrow.

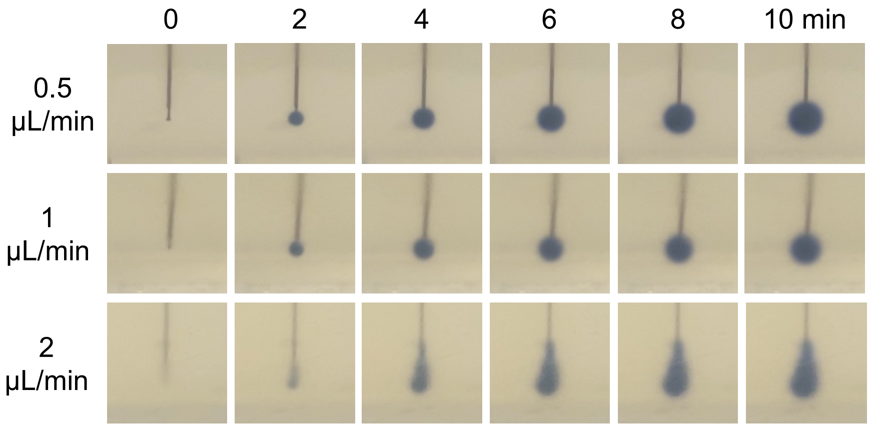

Figure S9. Evaluation of flow rates in 0.6 wt% agarose gel using Trypan blue as an indicator.

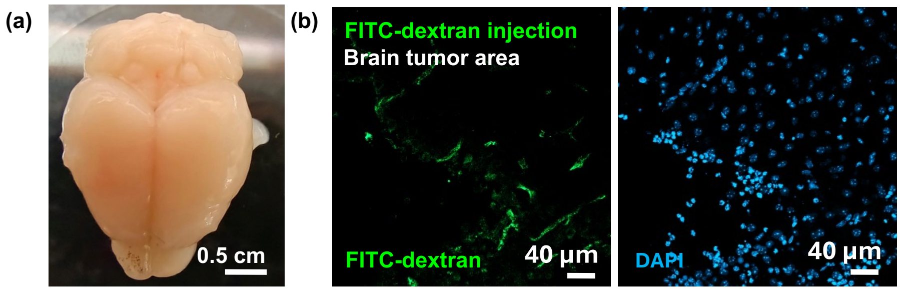

Figure S10. (a) The brain image after Evan's blue dye (2% in normal saline) administered intravenously (3 mL/kg) following the establishment of brain tumors in mice. (b) The CLSM images of brain after injection of 10,000 Da Fluorescein Isothiocyanate (FITC)-dextran.

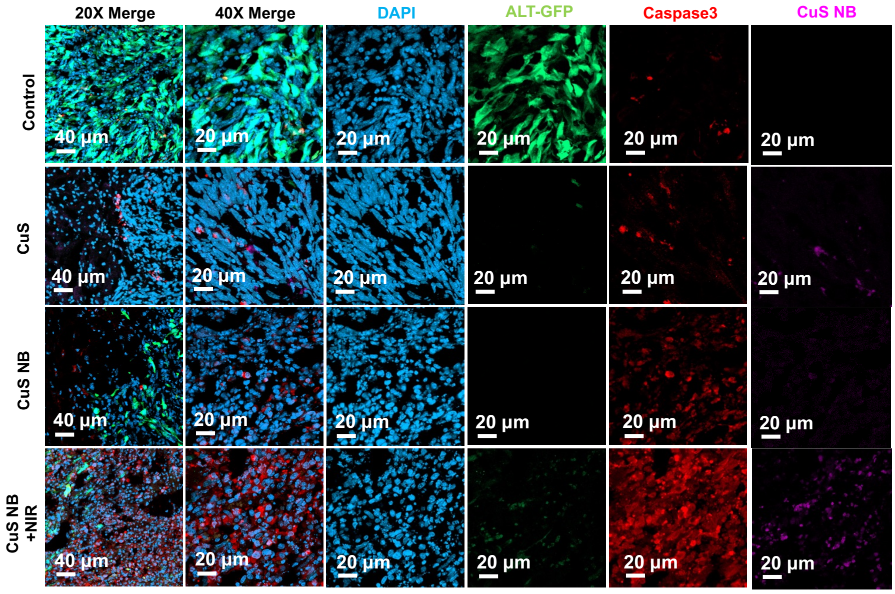

Figure S11. CLSM images depicting tumor slices subjected to various treatments. The cell nucleus, stained with DAPI, is represented in blue; ALTS1C1-GFP cells are displayed in green; and apoptosis, analyzed through cleaved-caspase 3, is presented in red.

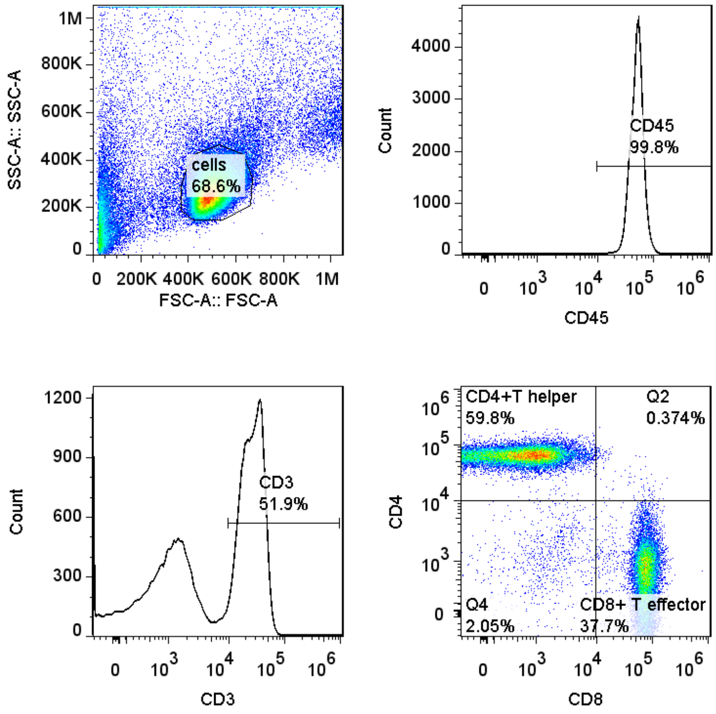

Figure S12. The gating strategy of flow cytometry.

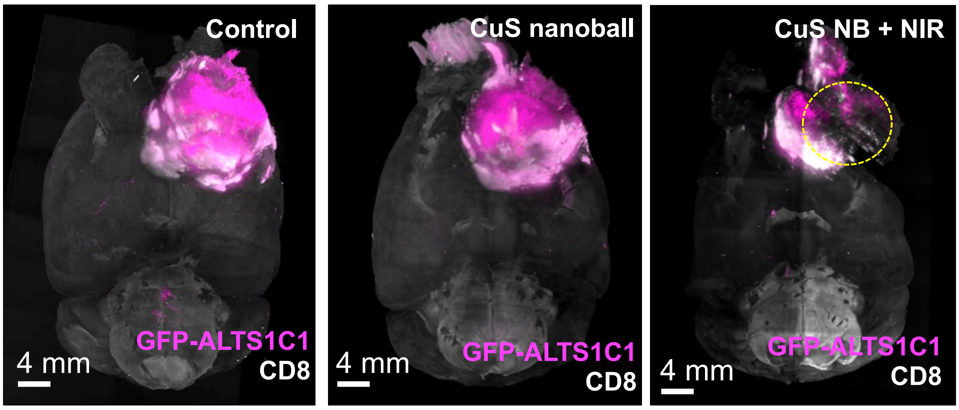

Figure S13. Whole brain images of GFP-ALTS1C1 brain tumor mice treated with CuS NB and CuS NB+NIR.

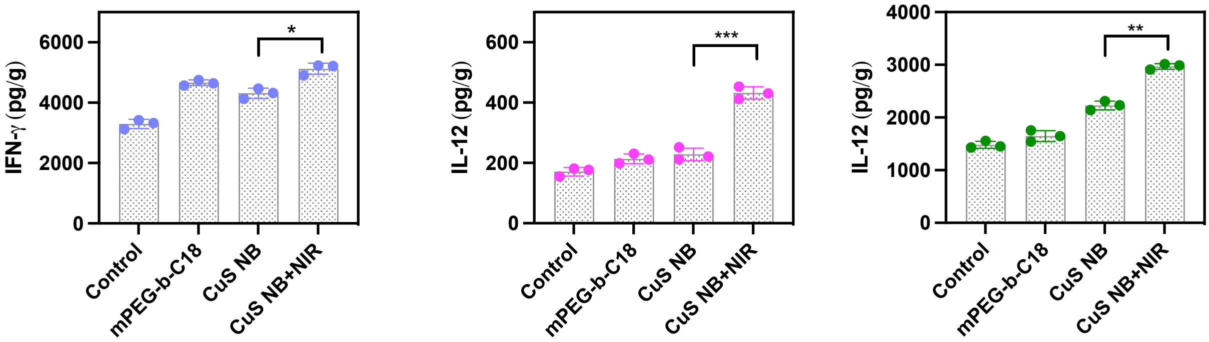

Figure S14. The concentrations of immune factors such as tumor necrosis factor-α (TNF-α), interferon-γ (IFN-γ), interleukin-10 (IL-10), and interleukin-12 (IL-12) in brain tissues treated with various samples were quantified using ELISA kits. (n = 3; mean ± s.d.; * $p < 0.05$; ** $p < 0.01$; *** $p < 0.05$; one-way ANOVA with Tukey's multiple comparison test).

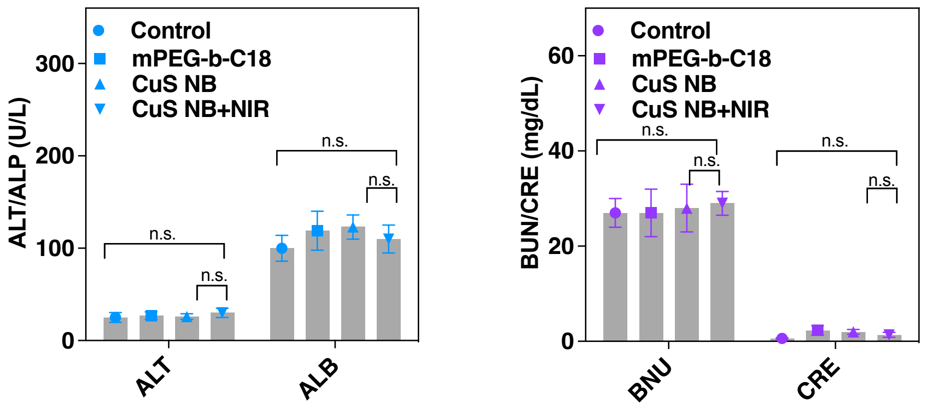

Figure S15. Biochemical indices of liver and kidney after 72 h of treatment (n = 3, mean ± s.d.; n.s.: no significant.)

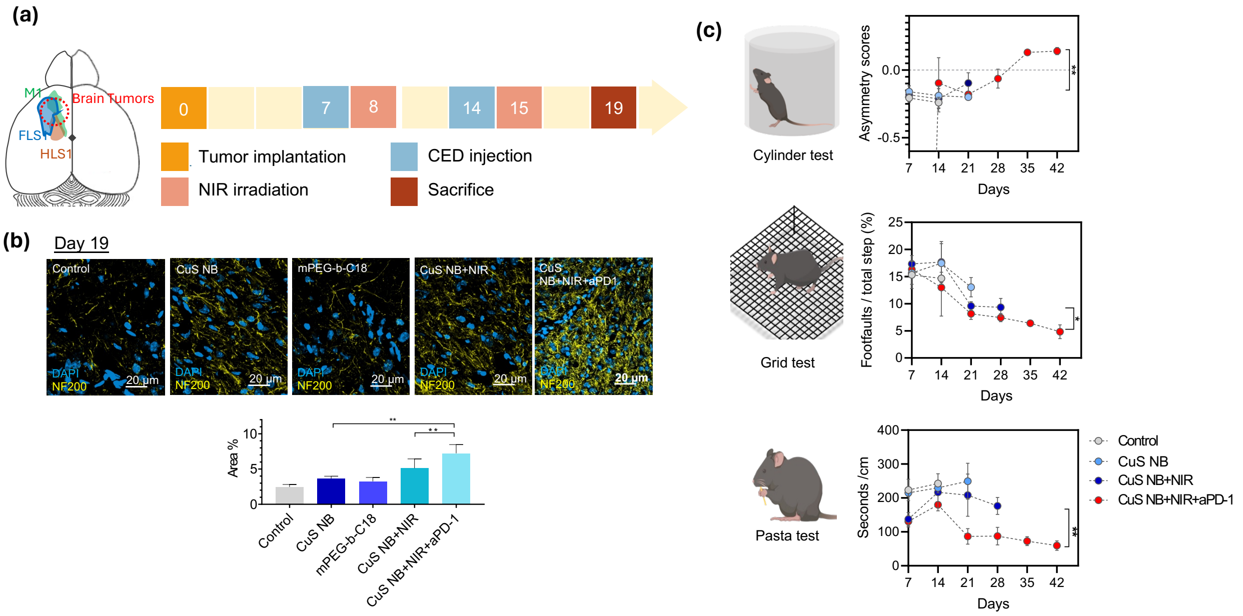

Figure S16. *In vivo* neural recovery and animal behavior in brain tumors treated with CuS NB, CuS NB+NIR, and CuS NB+NIR+aPD-1. (a) Brain tumor treatment options and subsequent analysis. (b) Confocal laser scanning microscopy (CLSM) image taken 19 days after injury showing neurofilament cells (stained purple with NF200) and blood vessels in the area adjacent to the injury site. Blue fluorescence represents DAPI-stained nuclei. (n = 5, mean ± s.d., *p < 0.05, **p < 0.01, one-way ANOVA with Tukey's multiple comparison test). (c) Behavioral tests after various conditions (cylinder, grid and pasta tests) (n = 5, mean ± s.d., *p < 0.05, **p < 0.01, one-way ANOVA with Tukey's multiple comparison test).
